# Associations of Immunological Proteins/Traits with Schizophrenia, Major Depression and Bipolar Disorder: A Bi-Directional Two-Sample Mendelian Randomization Study

**DOI:** 10.1016/j.bbi.2021.07.009

**Published:** 2021-07-16

**Authors:** Benjamin I. Perry, Rachel Upthegrove, Nils Kappelmann, Peter B. Jones, Stephen Burgess, Golam M. Khandaker

**Affiliations:** 1 Department of Psychiatry, University of Cambridge School of Clinical Medicine, Cambridge, England; 2 Cambridgeshire and Peterborough NHS Foundation Trust, Cambridge, England; 3 Institute for Mental Health, University of Birmingham, Birmingham, England; 4 Department of Translational Research in Psychiatry, Max Planck Institute of Psychiatry, Munich, Germany; 5 International Max Planck Research School for Translational Psychiatry (IMPRS-TP), Munich, Germany; 6 MRC Biostatistics Unit, University of Cambridge, Cambridge, England; 7 MRC Integrative Epidemiology Unit, Population Health Sciences, Bristol Medical School, University of Bristol, Bristol, England; 8 Centre for Academic Mental Health, Population Health Sciences, Bristol Medical School, University of Bristol, Bristol, England; 9 Avon and Wiltshire Mental Health Partnership NHS Trust, Bristol England

## Abstract

**Background:**

Schizophrenia, bipolar disorder and depression are associated with inflammation. However, it is unclear whether associations of immunological proteins/traits with these disorders are likely to be causal, or could be explained by reverse causality/residual confounding.

**Methods:**

We used bi-directional two-sample Mendelian randomization (MR) and multi-variable MR (MVMR) analysis to examine evidence of causality, specificity and direction of association of 20 immunological proteins/traits (pro-inflammatory cytokines: interleukin (IL)-6, tumour necrosis factor (TNF)-α, IL-12, IL-16, IL-17, IL-18; anti-inflammatory cytokines: IL-1 receptor antagonist (RA), IL-10, IL-13; chemokines: IL-8, monocyte chemo-attractant protein-1 (MCP-1); lymphoid growth-factors: soluble (s) IL-2Rα, IL-4, IL-7, IL-9; myeloid growth-factor: IL-5; acute phase protein: C-Reactive Protein (CRP); immune cells: neutrophils, lymphocytes; neurotrophic factor: brain derived neurotrophic factor (BDNF)) with schizophrenia, major depression and bipolar disorder.

**Results:**

Genetically-predicted IL-6 was associated with increased risk of schizophrenia in univariable MR (OR=1.24; 95% C.I., 1.05-1.47) and with major depression in MVMR (OR=1.08; 95% C.I., 1.03-1.12). These results survived Bonferroni-correction. Genetically-predicted sIL-2Rα (OR=1.07; 95% C.I., 1.01-1.12) and IL-9 (OR=1.06; 95% C.I., 1.01-1.11) were associated with increased schizophrenia risk. Genetically-predicted BDNF (OR=0.97; 95% C.I., 0.94-1.00) and MCP-1 (OR=0.96; 95% C.I., 0.91-0.99) were associated with reduced schizophrenia risk. However, these findings did not survive correction for multiple testing. The CRP-schizophrenia association attenuated completely after taking into account IL-6 and sIL-2Rα in MVMR (OR=1.02; 95% C.I., 0.81-1.28). No significant associations were observed for bipolar disorder. Evidence from bidirectional MR did not support reverse causality.

**Conclusions:**

We report evidence in support of potential causal associations of several immunological proteins/traits with schizophrenia, and of IL-6 with depression. Some of the findings did not survive correction for multiple testing and so replication in larger samples is required. Experimental studies are also required to further examine causality, mechanisms, and treatment potential for these immunological proteins/pathways for schizophrenia and depression.

## 1 Introduction

Schizophrenia, depression and bipolar disorder are serious mental disorders which overlap genetically and clinically (Anttila et al., 2018), indicative of shared aetiological mechanisms. Low-grade systemic inflammation/immune dysregulation could be one such mechanism (Goldsmith et al., 2016). Meta-analyses of cross-sectional studies have reported changes in concentrations of various immunological proteins/traits including inflammatory cytokines, acute phase proteins and other immune-regulatory proteins in blood (Goldsmith et al., 2016; Rodrigues-Amorim et al., 2018; Rowland et al., 2018) and cerebrospinal fluid (Wang and Miller, 2018) in these disorders. Longitudinal studies have reported associations between elevated levels of circulating inflammatory markers at baseline and risks of psychosis (Khandaker et al., 2014), depressive (Lamers et al., 2019) and bipolar (Hayes et al., 2017) disorders at follow-up, suggesting inflammation could be a cause rather than consequence of illness. While these studies have controlled for various confounders, residual confounding still remains an important alternative explanation. For example, autoimmune (Andersson et al., 2015; Eaton et al., 2006) and cardiometabolic disorders (Kendler et al., 2009; Perry et al., 2016) are often comorbid with schizophrenia and depression and may predispose to inflammation.

Mendelian randomization (MR) is an epidemiological approach that uses genetic variants (single nucleotide polymorphisms; SNPs) as proxies for a putative risk factor to untangle the problems of reverse causation (as genetic variants are fixed at conception, hence genetically-predicted levels of risk factors must precede any event) and unmeasured confounding (as genetic variants are often specific in their associations with risk factors) (Smith and Ebrahim, 2003). If genetically-predicted levels of a risk factor are associated with a disease outcome, then it is possible that the association between the risk factor and outcome has a causal basis. Existing MR studies have reported mixed findings regarding a potential causal role for inflammation in serious mental disorders. For instance, elevated CRP was reported to be protective for schizophrenia (Hartwig et al., 2017) but a risk factor for depression (Khandaker et al., 2020), while other studies have reported null findings for depression (Kappelmann et al., 2021; Wium-Andersen et al., 2014). Further work is needed to enable more definitive conclusions. For example, most previous MR studies have solely focussed on associations of IL-6 and CRP with mental disorders. Yet, considering a wider range of immunological proteins/traits may provide greater insights into system-level immunological changes, elucidate potential common/specific immune-related pathophysiologic mechanisms, and identify novel treatment targets.

We conducted two-sample MR analyses to test whether a range of immunological proteins/traits could be potentially causally associated with schizophrenia, major depression and bipolar disorder. We selected 20 immunological proteins/traits, including pro- and anti-inflammatory cytokines, acute phase proteins, chemokines, growth factors, and immune cells. Exposures were selected based on their associations with the psychiatric disorders in previous observational research (Frydecka et al., 2018; Goldsmith et al., 2016; Green et al., 2011; Rodrigues-Amorim et al., 2018; Rowland et al., 2018). In addition, we conducted bidirectional MR using immunological proteins/traits as outcomes to test the direction of association (i.e. whether psychiatric disorders may causally influence immune markers). We performed multi-variable-MR (MVMR) to estimate whether immunological proteins/traits are associated with psychiatric outcomes independently of each other and to explore potential interaction between immunological proteins/traits.

## 2 Methods and Materials

### 2.1 Selection of Genetic Variants Related to Immunological Proteins/Traits

We included: (i) pro-inflammatory cytokines: IL-6, tumour necrosis factor (TNF)-α, IL-12, IL-16, IL-17, IL-18; (ii) anti-inflammatory cytokines: IL-1 receptor antagonist (RA), IL-10, IL-13; (iii) chemokines: IL-8, monocyte chemo-attractant protein-1 (MCP-1); (iv) growth-factors for lymphoid cells: soluble (s) IL-2 receptor alpha subunit (sIL-2Rα), IL-4, IL-7, IL-9; (v) a growth-factor for myeloid cells: IL-5; (vi) an acute phase protein: CRP; (vii) immune cells: neutrophils, lymphocytes; and (viii) a neurotrophic growth factor with roles in inflammation (Jin et al., 2019): brain derived neurotrophic factor (BDNF). Summary statistics for genetic variants associated with these traits were obtained from large genome-wide association studies (GWAS) of European participants (Table 1). All GWAS adjusted for age, sex and population structure. Informed consent was sought for all participants per the original GWAS protocols.

For each trait, we used two sets of instruments (Supplementary Tables 1-2). First, we used all independent (10,000kb pairs apart, r^2^<0.001) SNPs reported to be associated with a trait (*p*<10^-5^) in the respective GWAS *(trans-*variants). Second, we selected a subset of SNPs that were located in the relevant coding gene region (*cis*-variants), based upon Genome Reference Consortium Human Build 37. While analysis of *trans*-variants provides a greater number of SNPs and increased statistical power, *cis-*variants are less likely than *trans* variants to be affected by pleiotropy (Swerdlow et al., 2016). For some traits (IL-4, IL-5, IL-7, IL-8, IL-9, IL-10, IL-13, IL-17, MCP-1, TNF-α, neutrophils, lymphocytes) we could not identify any *cis* variants at *p*<10^-5^, so these traits were excluded from *cis* analyses.

For IL-6, we included three *cis* instruments based on SNP effects on either circulating IL-6 levels or downstream effects on CRP levels. The Swerdlow *et al* and Sarwar *et al* instruments were based on variants in the *IL6R* region (Sarwar et al., 2012; Swerdlow et al., 2012). While these variants increase circulating IL-6 levels, the Sarwar *et al* instrument in particular *(IL6R* Asp358Ala; rs2228145 A>C) has been shown in a peripheral blood mononuclear cell-based experiment (Ferreira et al., 2013) to decrease CRP levels in carriers of the minor 358Ala allele (effect allele C) as a result of impaired IL-6 classical signalling, due to decreased expression of membrane-bound IL-6R (Ferreira et al., 2013). This variant is in high LD with the variants used in the Swerdlow *et al* instrument. Therefore, based on the existing literature, variants used in the Swerdlow *et al* and Sarwar *et al* instruments increase circulating IL-6 levels, yet downregulate IL-6 classical signalling, leading to decreased CRP levels. The third IL-6 instrument was based on a study by Georgakis *et al* (Georgakis et al., 2020), where SNPs in the *IL6R* region were coded to reflect higher circulating CRP levels, as a potential indicator of upregulated IL-6 signalling (Georgakis et al., 2020). For example, the *IL6R* variant rs2228145 features in both the Sarwar *et al* and Georgakis *et al* IL-6 instruments. However, the Sarwar *et al* instrument used the C allele as the effect allele (associated with lower CRP due to downregulated IL-6 classical signalling), but the Georgakis *et al* instrument used the A allele as the effect allele (associated with higher CRP levels due to upregulated IL-6 classical signalling). See Table 1 and Supplementary Figure 1.

Where a specific SNP was not available in the outcome dataset, we located proxy SNPs using linkage disequilibrium (LD) tagging (r^2^>0.8) via *LDlink* (Machiela and Chanock, 2015). Approximated F-statistics (beta^2^/se^2^) (Pierce et al., 2011) were calculated for each genetic instrument used for each exposure as a measure of instrument strength (Supplementary Tables 3-4). For bidirectional analyses, where possible we used complete summary data from the same GWAS used to derive genetic instruments for immunological proteins/traits as exposures. However, this was not possible for IL-6 or BDNF. For bidirectional analysis with IL-6 as the outcome we used data from *n*=8,293 European participants from a separate GWAS (Ahola-Olli et al., 2017). Bidirectional analysis for BDNF could not be conducted due to unavailability of full GWAS summary statistics.

### 2.2 Selection of Genetic Variants Related to Schizophrenia, Depression and Bipolar Disorder

We used summary statistics from recent GWAS from the Psychiatric Genomics Consortium (PGC) based on participants of European ancestry for schizophrenia (40,675 cases and 64,643 controls) (Pardinas et al., 2018), major depression (135,458 cases and 344,901 controls) (Wray et al., 2018) and bipolar disorder (41,917 cases and 371,549 controls) (Mullins et al., 2021). See Supplementary Methods for more information on the psychiatric outcome definitions and samples. Exposure and outcome GWAS were from different consortia, minimising the risk of bias from sample overlap. Power calculations for each psychiatric outcome are presented in Supplementary Table 5. For bidirectional analyses, we used independent genome-wide significant SNPs for schizophrenia (145 SNPs) (Pardinas et al., 2018), depression (44 SNPs) (Wray et al., 2018) and bipolar disorder (49 SNPs) (Mullins et al., 2021) as exposures.

### 2.3 Statistical Analysis

We obtained summary-level data from each GWAS including SNP rs identifier, β-coeffícient or log odds ratio, standard errors or 95% confidence intervals (CI), effect allele, other allele, *p*-value, effect allele frequency and sample size. MR was conducted using the *TwoSampleMR* package (Hemani et al., 2017; Hemani et al., 2018) for R (R Core Team, 2017). Instruments were clumped for linkage disequilibrium (LD) (i.e. where more than one SNP with potentially different effects is tagged by an exposure SNP) to ensure independence. For palindromic SNPs, the forward strand was inferred where possible using allele frequency information, and alleles were harmonised based on matching alleles.

Where ≥2SNPs were available, our primary MR method was inverse variance weighted (IVW) regression. We also conducted weighted median and MR-Egger regression (Supplementary Methods). Where a single SNP was available for an exposure, we used the Wald (ratio of coefficients) method.

Because of the genetic similarity between schizophrenia, bipolar disorder and depression (Anttila et al., 2018), and similarity in observational associations with immunological proteins/traits (Goldsmith et al., 2016; Rodrigues-Amorim et al., 2018; Rowland et al., 2018), we conducted multivariable MR (MVMR) analysis (Burgess and Thompson, 2015) on all three mental disorders for immunological proteins/traits showing evidence of univariable MR associations with at least one mental disorder. In MVMR, the associations for each genetic instrument of each exposure were conditioned on one another. To reduce the risk of violating MR assumptions, particularly that genetic instruments must not be associated with any confounder of the exposure-outcome association, we only considered MVMR for *cis* variants. IVW MVMR was performed using the *TwoSampleMR* package (Hemani et al., 2018).

For binary psychiatric outcomes, estimates represent log-odds ratios converted into odds ratios (ORs) and 95% CIs, representing the change in risk of psychiatric outcome per standard deviation (SD) increase in genetically-predicted levels of immunological protein/trait. For bi-directional MR, which estimated associations of schizophrenia, major depression and bipolar disorder (exposure; binary variable) with immunological proteins/traits (outcome; continuous variable), β-coeffícients and standard errors (SEs) represent the SD change in immunological protein/trait per unit increase in the log-odds of psychiatric disorder. The evidential threshold (*p*<0.05) was corrected for multiple testing based upon the number of exposures at each stage of analysis using the Bonferroni method (*p*<0.003 for analyses of *trans* variants (20 exposures), *p*<0.005 for analyses of *cis* variants (nine exposures); *p*<0.017 for MVMR analyses (three exposures)).

We performed several sensitivity analyses to estimate the robustness of our results. Heterogeneity among SNPs included in each analysis was estimated using the Cochran’s Q test. We explored horizontal pleiotropy (where an exposure SNP influences the outcome by mechanisms other than through the exposure) using the MR Egger regression intercept and the ‘MR pleiotropy residual sum and outlier’ (MR-PRESSO) method (Verbanck et al., 2018) (Supplementary Methods). Using MR-PRESSO, we performed the global test to estimate for horizontal pleiotropy, and where evident, used the method to correct the IVW-estimate via outlier removal. We estimated measurement error in SNP-exposure associations using the *I^2^_GX_* statistic (Bowden et al., 2016).

## 3 Results

### 3.1 Associations of Immunological Proteins/Traits with Schizophrenia, Major Depression and Bipolar Disorder

#### 3.1.1 Results for Univariable Mendelian Randomization

From analyses of *trans* variants (Figure 1; Supplementary Tables 6-8), each SD increase in genetically-predicted levels of IL-9 (IVW OR=1.06; 95% C.I., 1.01-1.11) increased schizophrenia risk, while each SD increase in levels of CRP (IVW OR=0.92; 95% C.I., 0.85-0.99) and MCP-1 (IVW OR=0.96; 95% C.I., 0.91-0.99) decreased schizophrenia risk. For each, the direction of effect was consistent across MR methods, although the evidence for each did not surpass the Bonferroni-corrected *p*-value threshold (*p*<0.003). There was no evidence for associations of *trans* variants with depression or bipolar disorder.

From analyses of *cis* variants (Figure 1; Supplementary Table 9), each SD increase in genetically-predicted levels of sIL-2Rα (Wald Ratio OR=1.07; 95% C.I., 1.01-1.12) increased schizophrenia risk, while each SD increase in levels of CRP (IVW OR=0.93; 95% C.I., 0.88-0.99) decreased schizophrenia risk. There was weak evidence that increased levels of BDNF decreased schizophrenia (Wald Ratio OR=0.97; 95% C.I., 0.94-1.00) and bipolar disorder (Wald Ratio OR=0.97; 95% C.I., 0.94-1.00) risk. However, the above associations of *cis* variants did not surpass the *Bonferroni*-corrected *p*-value threshold (*p*<0.005).

IL-6 was associated with schizophrenia using all three *cis* instruments. Per SD increase in genetically-predicted IL-6 levels, the OR for schizophrenia was: IVW OR=1.24 (95% C.I., 1.04-1.47) using the Swerdlow *et al* instrument; and Wald Ratio OR=1.10 (95% C.I., 1.03-1.17) using the Sarwar *et al* instrument. Using the Georgakis *et al* instrument, coded to reflect per SD increase in CRP levels associated with *IL-6R* variants, the OR for schizophrenia was IVW OR=0.72; 95% C.I., 0.58-0.88). For each, the direction of effect was consistent across MR methods, although only the association of IL-6 using the Georgakis *et al* instrument surpassed the *Bonferroni*-corrected *p*-value threshold (*p*<0.005). There was no evidence for associations of *cis* variants with depression or bipolar disorder.

#### 3.1.2 Results for Multivariable Mendelian Randomization

We included IL-6 (Swerdlow *et al* instrument), CRP and sIL-2Rα in MVMR, since these biomarkers were associated with schizophrenia in univariable MR. After taking into account CRP and sIL-2Rα, increased IL-6 levels were associated with increased risk for both schizophrenia (IVW OR=1.26; 95% C.I., 1.05-1.52) and major depression (IVW OR=1.08; 95% C.I., 1.03-1.12). sIL-2Rα remained associated with schizophrenia (IVW OR=1.05; 95% C.I., 1.01-1.10), but the CRP-schizophrenia association attenuated to the null. Except for the sIL-2Rα-schizophrenia association, all MVMR associations surpassed the *Bonferroni* corrected threshold (*p*<0.017) (Figure 2; Supplementary Table 10).

### 3.2 Test for Bi-directionality of Association using Psychiatric Disorders as Exposure

There was no evidence for potential causal effects of schizophrenia, major depression or bipolar disorder on any included immunological proteins/traits (Supplementary Tables 11-13) after correction for multiple testing. However, there was weak evidence for a protective effect of IL-13 (IVW β=-0.12, SE=0.06, *p*=0.063) and of IL-17 (IVW β=-0.11, SE=0.06, *p*=0.070) on the risk of bipolar disorder.

### 3.3 Results for Sensitivity Analyses

#### 3.3.1 Tests for Horizontal Pleiotropy

Using the MR Egger regression intercept test, there was no evidence of horizontal pleiotropy for *trans* analyses of any immune-regulatory protein/trait with either schizophrenia, major depression or bipolar disorder as outcomes (all *p*>0.05) (Supplementary Table 14). However, the MR-PRESSO global test suggested that horizontal pleiotropy may have affected *trans* analyses of IL-1RA, sIL-2Rα, IL-17, CRP, BDNF, neutrophils and lymphocytes with schizophrenia only (all *p*<0.05) (Supplementary Table 16). The association of CRP with schizophrenia (Outlier-Corrected IVW β=-0.11; SE 0.03) was preserved in MR-PRESSO outlier-corrected IVW analysis, but the associations of *trans* variants for sIL-2Rα and BDNF with schizophrenia attenuated (Supplementary Table 16).

In bidirectional analyses, using the MR Egger regression intercept test, there was evidence of horizontal pleiotropy affecting schizophrenia, major depression and bipolar disorder as exposures with neutrophils and lymphocytes as outcomes (all *p*<0.05) (Supplementary Table 15). The MR-PRESSO Global Test additionally suggested the potential of horizontal pleiotropy affecting schizophrenia with IL-12 and CRP as outcomes, bipolar disorder with CRP as outcome, and major depression with CRP as outcome (*p*<0.05) (Supplementary Table 17). Following outlier correction, there was evidence for a protective effect of CRP on risk of bipolar disorder (Outlier-Corrected IVW β=-0.03, SE=0.01).

#### 3.3.2 Test for Heterogeneity

There was evidence of heterogeneity for *trans* variants of IL-1RA, sIL-2Rα, IL-4, IL-5, IL-13, CRP, BDNF, neutrophils and lymphocytes with schizophrenia (all *p*<0.05), but no evidence for major depression or bipolar disorder as outcomes (all *p*>0.05 (Supplementary Table 14).

In bidirectional analyses, there was evidence of heterogeneity for bipolar disorder as exposure with sIL-2Rα, CRP, neutrophils and lymphocytes as outcomes, and major depression as exposure with CRP as outcome (all *p<*0.05) (Supplementary Table 15).

#### 3.3.3 Test for Measurement Error

Results for the *I^2^_GX_* tests indicated some evidence of measurement error, which may have biased MR Egger estimates for *trans* variants of IL-1RA and IL-17 as exposures. For bidirectional MR analyses, there was evidence of measurement error which may have affected each of schizophrenia, bipolar disorder and depression as exposures (Supplementary Table 18).

## 4 Discussion

We conducted MR analyses to test for evidence of potential causality and direction of association of a range of immunological proteins/traits in relation to schizophrenia, depression and bipolar disorder. First, our results indicate some evidence in support of a potential causal relationship between IL-6, IL-9, MCP-1, sIL-2Rα, and BDNF with schizophrenia, although only the association with IL-6 survived correction for multiple testing. Second, we report some evidence in support of a causal relationship between IL-6 and major depression after controlling for CRP and sIL-2Rα. Third, we show that the previously reported protective effect of CRP on schizophrenia (Hartwig et al., 2017; Lin et al., 2019) could be explained by IL-6, an upstream inducer of CRP production. Fourth, we found only weak evidence for a potential causal relationship between immunological proteins/traits with bipolar disorder, limited to BDNF, although the evidence did not meet the conventional or Bonferroni-corrected evidential thresholds. Finally, we found no evidence that schizophrenia or depression causally influence levels of any included immunological proteins/traits, and only weak evidence for a protective effect of bipolar disorder on CRP, IL-13 and IL-17 levels that did not meet the conventional or Bonferroni-corrected evidential thresholds, suggesting that reverse causality is an unlikely explanation for increased inflammation observed in patients with these disorders.

### 4.1.1 The potential role of IL-6 in schizophrenia and depression

Higher circulating IL-6 levels are associated with schizophrenia and depression in cross-sectional (Goldsmith et al., 2016) and longitudinal (Khandaker et al., 2014; Lamers et al., 2019) studies, and are associated with treatment resistance (Lin et al., 1998; Maes et al., 1997). In line with this evidence and results from previous MR studies (Hartwig et al., 2017; Khandaker et al., 2020), we report evidence for a potential causal role of IL-6 in schizophrenia, in both univariable MR and MVMR analyses. In addition, we report some evidence for a potential causal role of IL-6 in depression, although this was evident only in MVMR analysis.

We used three genetic instruments for IL-6, all comprising variants in/around the *IL6R* gene locus. The Swerdlow *et al* and Sarwar *et al* instruments coded SNPs to reflect higher circulating IL-6 levels. We report that these instruments were associated with increased schizophrenia risk. The Georgakis *et al* instrument coded SNPs to reflect higher circulating CRP levels. These SNPs were associated with decreased schizophrenia risk. These findings are intriguing because IL-6 is a key inducer of CRP production in hepatocytes (Hunter and Jones, 2015), and so one might expect both to be associated with illness risk in a similar way. One explanation for this divergence could be a potential differential role of IL-6 classic and trans-signalling with regards to schizophrenia risk.

Briefly, IL-6 classic signalling involves IL-6 binding to membrane-bound IL-6 receptors (IL-6Rs), which are only present on limited cell types. IL-6 trans-signalling involves cells that do not classically express IL-6Rs, but are induced if IL-6 binds with circulating soluble IL-6Rs (sIL-6R) to form IL-6-sIL-6R complexes, which, in turn, bind with the ubiquitous glycoprotein 130 (Hunter and Jones, 2015).

Comparing our MVMR findings to the biology of IL-6 might suggest that IL-6 trans-signalling could be the main driver of the risk-increase. This is because we found that genetically predicted increases in IL-6 were associated with an increased risk of schizophrenia and depression, while the association with genetically-predicted increased CRP fully attenuated in schizophrenia. This pattern of results indicates that CRP, and hence IL-6 classic signalling, is unlikely to be the main “causal driver” of schizophrenia risk.

The *IL6R* variant Asp358Ala (rs2228145; A>C), included in two instruments, is associated with increased circulating levels of IL-6 and sIL-6R but decreased CRP (Georgakis et al., 2020; Sarwar et al., 2012). A peripheral blood mononuclear cell-based experiment has reported that this variant impairs IL-6 classical signalling due to decreased expression of membrane-bound IL-6R (Ferreira et al., 2013). However, rs2228145 has divergent effects on various inflammatory illnesses, with the minor 358Ala allele conferring protection from coronary heart disease, rheumatoid arthritis (RA), and atrial fibrillation, but increased susceptibility to asthma (Ferreira et al., 2013). While we found a similar direction of effect of the minor allele on risks for schizophrenia and depression, the evidence was weaker in depression in univariable MR analysis, and may have been masked by genetic associations with CRP.

Regarding previous work, for context, in a separate study using depressive symptoms as outcome, we found the reverse pattern of association, whereby point estimates suggested a risk-increase for depression with the Georgakis *et al* instrument and a risk-decrease with Swerdlow *et al* and Sarwar *et al* instruments (Kappelmann et al., 2021), although the confidence intervals overlapped with our present findings. Additionally, studies in other illnesses using the Georgakis *et al* instrument have reported risk-increases for rheumatoid arthritis, cardiovascular disease (Georgakis et al., 2020), COVID-19 hospitalisation (Bovijn et al., 2020) and type 2 diabetes (Georgakis et al., 2020) as well as risk-decreases for bacterial infections and atopic/contact dermatitis (Georgakis et al., 2020). Therefore, IL-6/IL-6R alterations in schizophrenia may be more closely aligned to vulnerability to infection as well as atopic/contact dermatitis, while IL-6/IL-6R alterations in depression may be more closely aligned to that found in rheumatoid arthritis and cardiovascular disease.

Nevertheless, disentangling the implications of IL-6/IL-6R signalling in psychiatric and non-psychiatric illnesses using population genomics is complex. This is because the functional roles of all individual genetic variants used as MR instruments are unknown (Garbers and Rose-John, 2021). In addition, IL-6 has a diverse repertoire of physiological functions including but not limited to angiogenesis (Barnes et al., 2011) and neurodevelopment (Rothaug et al., 2016). While the MR approach can provide evidence for potential causality, we cannot disentangle the effects of trans vs classical signalling, mechanisms for divergent effects of classical signalling on various inflammatory diseases, or the non-immune functions of IL-6, based on MR findings alone. Therefore, experimental human and animal studies that directly modulate individual IL-6 pathways are required to triangulate the potential causal mechanisms of IL-6 in schizophrenia and in depression. Ultimately, this could support the development of more targeted immune-modulating interventions for psychiatric disorders such as schizophrenia and depression.

### 4.1.2 Clinical Implications

Our results indicate that the IL-6/IL-6R pathway may represent a novel therapeutic target for schizophrenia and depression. IL-6 could also be a shared mechanism for cardiovascular disease (Khandaker et al., 2020), which is often comorbid with depression (Kendler et al., 2009) and is also associated with IL-6 (Danesh et al., 2008). However, a recent RCT found no effect of tocilizumab for established schizophrenia (Girgis et al., 2018). Tocilizumab is an anti-IL-6R monoclonal antibody (mAb) which blocks IL-6 classic and trans signalling and is licensed for RA. The null findings may be due to patient selection, and patients with evidence of immuno-activation at baseline may be better candidates for immunotherapy (Raison et al., 2013). A recent RCT of anti-IL-6 mAb sirukumab found improvement in anhedonia in depressed patients selected based on elevated CRP (NCT02473289). We are now conducting a proof-of-concept RCT of tocilizumab for depressed patients with elevated CRP and somatic symptoms of depression (Khandaker et al., 2018). Further clinical trials of immunotherapies including that of anti-IL-6 drugs along with careful patient selection are required.

### 4.2 Associations of other immune biomarkers with psychiatric disorders

Whilst several of the MR associations did not survive correction for multiple testing, the overall pattern of evidence highlights the importance of considering immune markers as part of a network/system rather than in isolation. We report that CRP is unlikely to be causally related to schizophrenia and depression. This is because a protective effect of CRP on schizophrenia risk, as reported previously (Hartwig et al., 2017), fully attenuated in MVMR analysis after accounting for the effects of IL-6 and sIL-2Rα, and we didn’t find evidence for an association of CRP with depression, in line with other MR evidence (Kappelmann et al., 2021). However, we found evidence, after outlier correction, for a potential protective effect of bipolar disorder on CRP levels in bidirectional analysis. This finding contradicts meta-analytic observational evidence suggesting that bipolar disorder is associated with higher CRP, particularly in states of euthymia and mania (Rowland et al., 2018). Our results suggest that confounding could be responsible for the observational findings. However, the finding did not surpass the Bonferroni-corrected evidential threshold and so should be considered with caution.

We found weak MR evidence for a small protective effect of BDNF on schizophrenia and bipolar disorder in analyses of *cis* variants only, consistent with previous meta-analyses (Green et al., 2011; Rowland et al., 2018). However, the evidence did not surpass conventional or Bonferroni-corrected evidential thresholds, and the *cis* instrument may have been affected by weak-instrument bias, possibly due to the relatively small GWAS for BDNF (Terracciano et al., 2013). In future, larger GWAS of BDNF may help to clarify these findings.

We found weak evidence for a potential causal relationship between sIL-2Rα and schizophrenia, consistent with a meta-analysis reporting elevated sIL-2R levels in schizophrenia (Goldsmith et al., 2016) and with epidemiological evidence linking autoimmune disorders with schizophrenia (Eaton et al., 2006). IL-2 is associated with control of T-cell function and autoimmunity, and over-activity of IL-2 is thought to contribute to the development of classical autoimmune conditions (Sharma et al., 2011). Therefore, the IL-2/IL-2R pathway could be a mechanistic link between schizophrenia and autoimmune conditions, and a potential therapeutic target for schizophrenia.

We found weak evidence for potential causal associations of MCP-1 and IL-9 with schizophrenia. MCP-1 is a potent pro-inflammatory chemokine involved in monocyte migration and infiltration (Yoshimura, 2018). However, our findings contrast meta-analytic reports of increased MCP-1 levels in first-episode psychosis (Frydecka et al., 2018). In future, a larger GWAS for MCP-1 is required to identify a larger number of variants, particularly *cis* variants, to clarify these findings. IL-9, a regulator of haematopoietic cells, is associated with childhood asthma (Koch et al., 2017). Asthma is associated with risk of adult psychotic disorders (Wu et al., 2019). IL-9 has been found to be associated with multi-episode schizophrenia in a cross-sectional study (Frydecka et al., 2018). Further investigation of this pathway could help to elucidate its role in schizophrenia and in comorbidity between schizophrenia and atopic disorders.

### 4.3 Strengths and Limitations

A key strength of our work is the use of a wide range of immunological proteins/traits, which permitted an examination of the wider inflammatory/immune network in three major psychiatric disorders. We used several MR techniques including tests for direction of association using bidirectional MR, interaction between biomarkers using MVMR, and assessed the robustness of our approach using detailed sensitivity analyses. The use of both *cis* and *trans* variants helped to address the potential pleiotropic effects of genetic variants.

Limitations of the work include relatively small GWAS for some exposures, although statistical power for MR is largely driven by the strength of SNP-outcome association (Pierce and Burgess, 2013). Nevertheless, analyses of *trans* instruments for a number of inflammatory exposures may have been affected by bias from heterogeneity and horizontal pleiotropy. Furthermore, statistical power calculations indicated that the analyses for all three psychiatric disorders may have been underpowered to detect particularly subtle causal effects. While a larger GWAS for depression exists (Howard et al., 2019), we did not include it due to the less-stringent diagnostic criteria for depression, which may have reduced phenotypic specificity (Cai et al., 2020). Also, we could not test associations with particular types of cases, e.g., first-episode or treatment resistant, due to the lack of detailed phenotype data in existing GWAS. Therefore, replication of our analyses using larger GWAS including those with more granular data on illness phenotypes would be useful. We used a *p*<10^-5^ cut-off for selection of trans instruments. Although this can increase the risk of weak-instrument bias for individual genetic variants, our F-statistics suggested that these instruments had appropriate strength (all >10), so lowering the *p*-value threshold should have resulted in an overall increase in statistical power for MR analysis. In future, when larger GWAS for inflammatory markers are available, more stringent selection criteria may be considered. Also, we could not address the relevance of timing for the effect of immunological proteins/traits on psychiatric outcomes, as the exposures were measured across different ages in adults. In future, larger GWAS may permit stratification of analyses performed at different timepoints to investigate the potential relevance of developmental vulnerability periods. Finally, all GWAS were based on European samples which reduced the risk of population stratification bias, but may limit the generalizability of findings to other ethnic groups. Many of our results did not surpass stringent thresholds for multiple testing, so replication of these MR findings using larger GWAS is required.

### 4.4 Conclusions

Our results indicate some evidence for a potential causal role of a number of immunological proteins/traits in schizophrenia including IL-6, sIL-2Rα, IL-9, MCP-1 and BDNF. IL-6 may also be causally linked to major depression. These markers may represent novel targets for treatment/prevention of these disorders. We found little evidence that psychiatric disorders causally influence immunological proteins/traits. In future, replication of these findings using even larger and more phenotypically granular GWAS are required. In addition, the field requires experimental studies to further examine causality, mechanisms, therapeutic potential, and the possible role of immunological proteins/traits for comorbidities of schizophrenia with autoimmune conditions and cardiovascular disease.

## Supplementary Material

Supplementary material

## Figures and Tables

**Figure 1:** ORs (95% CIs) from Mendelian Randomization Analysis Showing Associations between Genetically Predicted Levels of Inflammatory Markers and Risks for Schizophrenia, Depression and Bipolar Disorder.

**Figure 2:** ORs (95% CIs) from MVMR Analysis Showing Associations between Genetically Predicted Levels of Inflammatory Markers and Schizophrenia, Depression and Bipolar Disorder.

**Table 1:** Immunological Proteins/Traits and Source of GWAS Summary Statistics

Group	Immune-Regulatory Proteins	GWAS for Summary Statistics and Sample Size
Pro-Inflammatory Cytokine	IL-6, TNF-α, IL-12, IL-16, IL-17, IL-18	IL-6: *n*=133,449 (Swerdlow et al., 2012)^a^; *n*=125,222 (Sarwar et al., 2012)^a^; *n=200,402* (Georgakis et al., 2020)^b^; All other: *n*=8,293 (Ahola-Olli et al., 2017)
Anti-Inflammatory Cytokine	IL-1RA, IL-10, IL-13	*n*=8,293 (Ahola-Olli et al., 2017)
Chemokine	IL-8, MCP-1	*n*=8,293 (Ahola-Olli et al., 2017)
Acute-Phase Protein	CRP	*n*=200,402 (Ligthart et al., 2018)
Growth Factor for Lymphoid Cells	sIL-2Rα, IL-4, IL-7, IL-9	*n*=8,293 (Ahola-Olli et al., 2017)
Growth Factor for Haematopoietic and Myeloid Cells	IL-5	*n*=8,293 (Ahola-Olli et al., 2017)
Immune Cell	Neutrophils, Lymphocytes	*n*=173,480 (Astle et al., 2016)
Neurotrophic Growth Factor	BDNF	*n*=2,054 (Terracciano et al., 2013)

a These genetic instruments for IL-6 were obtained from Swerdlow *et al* (Swerdlow et al., 2012) and Sarwar *et al* (Sarwar et al., 2012), and are based on SNPs in the *IL6R* gene region. These variants are associated with increased circulating IL-6 levels.

b This genetic instrument for IL-6 was obtained from Georgakis *et al* (Georgakis et al., 2020), which is also based on SNPs in the *IL6R* gene region. However, Georgakis *et al* coded their data to reflect associations of these variants with increased circulating CRP levels as a downstream readout of IL-6 activity. We used the same coding for our analysis.
