# Supplementary material for: Associations of Immunological Proteins/Traits with Schizophrenia, Major Depression and Bipolar Disorder: A Bi-Directional Two-Sample Mendelian Randomization Study
Source: Brain Behav Immun. Author manuscript; Available in PMC 2022 Jul 2. (PMC7612947; doi:10.1016/j.bbi.2021.07.009)
Supplement: Supplementary material [file EMS146248-supplement-Supplementary_material.docx]

**Online Supplementary Data**

**Perry *et al.* Associations of Immunological Proteins/Traits with Schizophrenia, Major Depression and Bipolar Disorder: A Bi-Directional Two-Sample Mendelian Randomization Study**

**Supplementary Table 1: Genetic variants included in *trans* analyses**

| **Immune-Regulatory Protein** | **SNP** | **Effect Allele** | **F-Statistic^a^** |
| --- | --- | --- | --- |
| IL-1RA | rs6699436 | G | 20.14 |
|  | rs12121840 | C | 22.23 |
|  | rs74949421 | G | 19.49 |
|  | rs4441609 | C | 20.06 |
|  | rs56134659 | G | 22.21 |
|  | rs35803309 | T | 26.51 |
|  | rs1054402 | C | 23.58 |
|  | rs74435620 | G | 20.76 |
|  | rs2809154 | C | 21.19 |
|  | rs11627423 | C | 22.48 |
|  | rs61335305 | C | 24.05 |
|  | rs12051139 | C | 19.70 |
|  | rs11869294 | G | 23.02 |
|  | rs2019023 | T | 24.29 |
|  | rs139005642 | G | 22.54 |
|  | rs9623661 | C | 21.30 |
| sIL-2Rα | rs929551 | G | 22.94 |
|  | rs185231391 | C | 22.09 |
|  | rs759244 | T | 22.20 |
|  | rs77313297 | C | 20.61 |
|  | rs62305210 | G | 20.42 |
|  | rs61705228 | C | 21.28 |
|  | rs115360066 | G | 24.27 |
|  | rs11241559 | G | 22.58 |
|  | rs28441585 | T | 19.66 |
|  | rs4733117 | C | 21.98 |
|  | rs12722497 | C | 167.61 |
|  | rs73001149 | C | 20.51 |
|  | rs12799226 | C | 20.44 |
|  | rs117026681 | G | 21.03 |
|  | rs72767608 | C | 19.62 |
|  | rs56192581 | G | 20.47 |
|  | rs117244812 | G | 22.54 |
|  | rs623264 | G | 19.94 |
|  | rs73910487 | G | 19.51 |
| IL-4 | rs116705532 | G | 22.88 |
|  | rs79597994 | C | 21.08 |
|  | rs12091966 | G | 20.38 |
|  | rs4444693 | T | 22.93 |
|  | rs11709228 | G | 19.99 |
|  | rs7613691 | G | 21.37 |
|  | rs13106889 | T | 26.63 |
|  | rs78876292 | C | 19.71 |
|  | rs73023729 | G | 24.08 |
|  | rs75924875 | G | 20.30 |
|  | rs6969391 | C | 20.69 |
|  | rs17713451 | G | 25.36 |
|  | rs10512267 | C | 26.19 |
|  | rs117146485 | C | 21.61 |
|  | rs9508291 | C | 21.80 |
|  | rs2073438 | G | 19.91 |
|  | rs2849346 | G | 21.78 |
|  | rs9941733 | G | 24.78 |
| IL-5 | rs116264291 | G | 19.42 |
|  | rs148634917 | G | 21.05 |
|  | rs6737109 | C | 22.06 |
|  | rs11680908 | G | 22.61 |
|  | rs7578892 | G | 22.19 |
|  | rs12635407 | G | 20.28 |
|  | rs72831687 | G | 22.32 |
|  | rs7767396 | G | 37.93 |
|  | rs10454319 | G | 19.83 |
|  | rs28793375 | C | 20.84 |
|  | rs71438922 | G | 19.94 |
|  | rs74811276 | G | 20.53 |
|  | rs144467962 | G | 19.40 |
|  | rs73040130 | C | 24.87 |
| IL-7 | rs12117072 | T | 20.79 |
|  | rs6707510 | G | 19.73 |
|  | rs75904417 | C | 23.67 |
|  | rs218247 | G | 22.35 |
|  | rs115215018 | C | 21.11 |
|  | rs141425475 | C | 22.14 |
|  | rs8175379 | T | 21.03 |
|  | rs4320361 | G | 169.84 |
|  | rs28793375 | C | 20.59 |
|  | rs117509142 | C | 22.59 |
|  | rs11774096 | T | 19.72 |
|  | rs78346957 | G | 20.76 |
|  | rs2095309 | G | 20.05 |
|  | rs17091524 | C | 23.63 |
|  | rs62006410 | C | 26.41 |
|  | rs77981494 | C | 23.68 |
|  | rs11662005 | G | 19.37 |
|  | rs144701438 | G | 23.74 |
|  | rs147747784 | G | 28.23 |
| IL-8 | rs12075 | G | 25.85 |
|  | rs116726256 | C | 19.79 |
|  | rs17866606 | C | 19.57 |
|  | rs141926526 | C | 22.10 |
|  | rs79274420 | G | 20.50 |
|  | rs2552220 | G | 19.92 |
|  | rs2673604 | C | 24.65 |
|  | rs7132236 | G | 20.59 |
|  | rs7135809 | T | 19.80 |
|  | rs144929914 | G | 19.75 |
|  | rs11634944 | C | 23.21 |
|  | rs2651759 | G | 19.87 |
|  | rs75840288 | C | 19.93 |
|  | rs3786107 | G | 20.41 |
|  | rs200282774 | C | 21.39 |
|  | rs12960757 | G | 20.46 |
| IL-9 | rs41294750 | C | 22.07 |
|  | rs17317275 | G | 20.19 |
|  | rs73443903 | C | 20.50 |
|  | rs73218490 | C | 20.11 |
|  | rs187422429 | C | 20.60 |
|  | rs10982712 | C | 20.40 |
|  | rs72819454 | C | 20.24 |
|  | rs4880409 | C | 21.53 |
|  | rs61867538 | C | 21.23 |
|  | rs76963786 | C | 26.46 |
|  | rs3736858 | G | 21.99 |
|  | rs186112202 | G | 20.32 |
|  | rs117807175 | G | 21.35 |
|  | rs61335305 | C | 19.53 |
|  | rs7242404 | G | 21.64 |
|  | rs7232268 | G | 22.09 |
|  | rs115201696 | C | 20.24 |
| IL-10 | rs11206302 | C | 22.44 |
|  | rs10493718 | C | 24.55 |
|  | rs3002131 | G | 20.67 |
|  | rs282258 | C | 37.50 |
|  | rs1530455 | C | 20.77 |
|  | rs6799107 | C | 20.60 |
|  | rs111913416 | T | 24.42 |
|  | rs2086656 | C | 21.29 |
|  | rs4345303 | C | 20.79 |
|  | rs3025021 | C | 23.58 |
|  | rs4349809 | G | 298.98 |
|  | rs41282660 | G | 21.92 |
|  | rs10457128 | G | 25.29 |
|  | rs2375980 | G | 24.94 |
|  | rs7088799 | G | 26.03 |
|  | rs77258639 | C | 19.49 |
|  | rs9951418 | C | 20.23 |
|  | rs465757 | G | 23.31 |
|  | rs9981861 | C | 19.62 |
| IL-12 | rs282258 | C | 21.90 |
|  | rs35473497 | G | 20.00 |
|  | rs6532374 | C | 19.97 |
|  | rs41282644 | G | 23.48 |
|  | rs4349809 | G | 564.29 |
|  | rs34467391 | G | 42.17 |
|  | rs13209117 | G | 29.02 |
|  | rs10958542 | C | 19.64 |
|  | rs6993770 | T | 23.59 |
|  | rs2375980 | G | 34.73 |
|  | rs199840084 | C | 22.73 |
|  | rs144160960 | C | 20.30 |
|  | rs7088799 | G | 38.42 |
|  | rs79121401 | C | 21.16 |
|  | rs782107 | G | 23.11 |
|  | rs12148636 | T | 19.78 |
|  | rs4886671 | C | 20.56 |
|  | rs72831623 | G | 26.73 |
|  | rs1996152 | C | 19.77 |
|  | rs273702 | G | 20.62 |
|  | rs71361173 | G | 21.57 |
|  | rs200751 | G | 21.23 |
|  | rs17229494 | G | 20.80 |
|  | rs137921 | C | 19.82 |
| IL-13 | rs75383097 | G | 21.17 |
|  | rs12623722 | G | 21.10 |
|  | rs75904417 | C | 19.85 |
|  | rs6799107 | C | 23.50 |
|  | rs75954784 | C | 20.18 |
|  | rs139083458 | C | 22.09 |
|  | rs27949 | C | 21.48 |
|  | rs72772212 | C | 19.60 |
|  | rs29545 | C | 20.49 |
|  | rs17722688 | C | 19.84 |
|  | rs75995699 | G | 22.61 |
|  | rs9472168 | G | 292.85 |
|  | rs142167313 | C | 25.73 |
|  | rs73322775 | G | 20.90 |
|  | rs117795020 | G | 24.20 |
|  | rs10995614 | C | 20.63 |
|  | rs7073807 | C | 22.32 |
|  | rs7910571 | T | 20.53 |
|  | rs78346957 | G | 20.09 |
|  | rs17647312 | C | 19.86 |
|  | rs147747784 | G | 22.85 |
|  | rs76339001 | T | 23.70 |
| IL-16 | rs4513633 | C | 24.43 |
|  | rs4253283 | C | 31.05 |
|  | rs1801020 | G | 40.59 |
|  | rs11787273 | C | 20.58 |
|  | rs115981497 | T | 24.41 |
|  | rs12765671 | G | 20.88 |
|  | rs1255143 | C | 29.12 |
|  | rs117916513 | G | 25.92 |
|  | rs9706053 | C | 24.17 |
|  | rs4778636 | G | 131.98 |
|  | rs144691581 | G | 25.49 |
|  | rs117217798 | C | 21.03 |
|  | rs72987269 | C | 20.33 |
|  | rs140996761 | G | 20.16 |
| IL-17 | rs57920188 | G | 20.48 |
|  | rs12735700 | G | 20.43 |
|  | rs78296352 | G | 21.96 |
|  | rs1446499 | C | 19.51 |
|  | rs17282552 | C | 24.41 |
|  | rs141312283 | G | 19.56 |
|  | rs1530455 | C | 38.97 |
|  | rs78612928 | C | 21.82 |
|  | rs187475560 | C | 21.91 |
|  | rs145577605 | G | 19.85 |
|  | rs148562661 | G | 23.93 |
|  | rs149738638 | C | 20.12 |
|  | rs117029961 | G | 20.41 |
|  | rs184080173 | C | 25.62 |
|  | rs9568764 | G | 19.58 |
|  | rs117556572 | C | 21.55 |
|  | rs17106604 | C | 25.18 |
|  | rs11640734 | G | 24.26 |
|  | rs62191444 | G | 21.15 |
|  | rs36045997 | G | 19.55 |
| IL-18 | rs385076 | C | 96.17 |
|  | rs13065560 | G | 19.73 |
|  | rs1656939 | T | 23.01 |
|  | rs4482818 | G | 27.78 |
|  | rs4414903 | G | 21.57 |
|  | rs116383510 | C | 26.40 |
|  | rs115267715 | C | 31.75 |
|  | rs17229943 | C | 45.41 |
|  | rs658805 | G | 25.25 |
|  | rs62425821 | G | 19.79 |
|  | rs117266781 | C | 21.72 |
|  | rs1852105 | C | 21.10 |
|  | rs78623212 | C | 23.97 |
|  | rs144841621 | C | 20.61 |
|  | rs2729385 | G | 22.08 |
|  | rs71478720 | C | 93.51 |
|  | rs4885797 | G | 19.78 |
|  | rs1979967 | C | 24.03 |
|  | rs143370787 | G | 22.29 |
|  | rs10414578 | C | 25.60 |
|  | rs78716465 | G | 22.92 |
|  | rs11700536 | C | 21.38 |
| Lymphocytes | rs4319365 | G | 35.76 |
|  | rs146490475 | C | 39.49 |
|  | rs41287280 | G | 81.67 |
|  | rs2157691 | C | 64.75 |
|  | rs3748022 | T | 64.19 |
|  | rs72781680 | T | 174.27 |
|  | rs1260326 | T | 49.38 |
|  | rs149290349 | A | 108.44 |
|  | rs990171 | A | 65.37 |
|  | rs7576541 | C | 82.58 |
|  | rs55706446 | T | 131.57 |
|  | rs2019137 | A | 43.83 |
|  | rs79716587 | A | 73.15 |
|  | rs113678689 | C | 93.37 |
|  | rs6433895 | C | 158.13 |
|  | rs6735656 | G | 81.52 |
|  | rs11676298 | G | 39.16 |
|  | rs58106596 | A | 51.73 |
|  | rs1822534 | G | 102.78 |
|  | rs6801231 | A | 51.56 |
|  | rs4502545 | A | 58.50 |
|  | rs13063578 | A | 222.19 |
|  | rs35592432 | C | 84.68 |
|  | rs59107033 | T | 41.02 |
|  | rs7650602 | C | 62.88 |
|  | rs13315469 | G | 33.21 |
|  | rs13092376 | C | 93.98 |
|  | rs1386622 | T | 323.88 |
|  | rs12504282 | C | 97.01 |
|  | rs6832951 | G | 83.26 |
|  | rs5026472 | C | 137.42 |
|  | rs62355272 | A | 190.58 |
|  | rs79237520 | T | 50.21 |
|  | rs2326837 | G | 46.05 |
|  | rs9658111 | C | 55.73 |
|  | rs72928038 | A | 52.56 |
|  | rs6568490 | T | 35.44 |
|  | rs516805 | G | 67.76 |
|  | rs9494142 | C | 93.53 |
|  | rs2347784 | G | 42.00 |
|  | rs13242809 | A | 42.05 |
|  | rs3735485 | A | 99.42 |
|  | rs13230583 | C | 38.25 |
|  | rs11250079 | C | 35.22 |
|  | rs4737010 | A | 51.76 |
|  | rs2919917 | C | 138.08 |
|  | rs11779535 | A | 38.13 |
|  | rs10087240 | T | 43.34 |
|  | rs6475611 | A | 107.92 |
|  | rs10867411 | A | 54.46 |
|  | rs1014530 | T | 42.10 |
|  | rs7090504 | A | 62.65 |
|  | rs10828723 | C | 69.17 |
|  | rs17011715 | G | 76.62 |
|  | rs748113 | C | 86.62 |
|  | rs7939778 | A | 40.86 |
|  | rs117706999 | A | 37.16 |
|  | rs6589939 | G | 121.88 |
|  | rs4937334 | A | 47.19 |
|  | rs34038797 | G | 40.81 |
|  | rs10466905 | A | 245.88 |
|  | rs10844706 | A | 337.40 |
|  | rs11168249 | C | 40.25 |
|  | rs17041439 | C | 40.49 |
|  | rs3184504 | T | 606.36 |
|  | rs28577594 | G | 51.72 |
|  | rs73579370 | C | 39.27 |
|  | rs696 | T | 52.41 |
|  | rs2281603 | C | 49.90 |
|  | rs175714 | T | 44.05 |
|  | rs2224234 | C | 61.20 |
|  | rs3540 | A | 95.71 |
|  | rs12598529 | G | 33.33 |
|  | rs12716977 | T | 264.97 |
|  | rs7192652 | G | 58.54 |
|  | rs247826 | T | 99.70 |
|  | rs6502555 | C | 72.70 |
|  | rs9913156 | C | 61.71 |
|  | rs3818717 | T | 50.07 |
|  | rs2228015 | C | 33.91 |
|  | rs11077815 | T | 44.06 |
|  | rs36084354 | A | 89.78 |
|  | rs5498 | G | 128.84 |
|  | rs7251806 | C | 597.14 |
|  | rs61387190 | T | 50.78 |
|  | rs3745621 | G | 67.59 |
|  | rs4618126 | A | 173.25 |
|  | rs1883932 | A | 48.10 |
|  | rs4911253 | G | 36.11 |
|  | rs6122940 | T | 35.64 |
|  | rs259981 | A | 41.63 |
|  | rs1297255 | T | 40.88 |
|  | rs2070433 | A | 37.61 |
|  | rs2073748 | A | 41.91 |
|  | rs5754100 | C | 99.60 |
|  | rs714027 | A | 89.35 |
| Neutrophils | rs12752838 | A | 43.52 |
|  | rs146091102 | A | 33.34 |
|  | rs3917932 | C | 172.54 |
|  | rs34293785 | C | 160.85 |
|  | rs663045 | G | 42.46 |
|  | rs34599082 | T | 126.65 |
|  | rs2208568 | T | 126.12 |
|  | rs12239046 | T | 80.63 |
|  | rs1260326 | T | 82.32 |
|  | rs113542380 | A | 44.85 |
|  | rs72917514 | A | 41.51 |
|  | rs10171849 | C | 87.80 |
|  | rs10173538 | T | 49.90 |
|  | rs6740847 | A | 47.87 |
|  | rs114050631 | T | 74.28 |
|  | rs9287604 | G | 56.14 |
|  | rs9872570 | A | 41.22 |
|  | rs6782228 | C | 70.11 |
|  | rs1567558 | A | 43.56 |
|  | rs11734460 | T | 45.55 |
|  | rs11931598 | T | 37.94 |
|  | rs218265 | C | 69.48 |
|  | rs1352846 | G | 40.66 |
|  | rs11725704 | G | 387.83 |
|  | rs7679673 | A | 40.65 |
|  | rs7705526 | A | 83.07 |
|  | rs11741826 | C | 62.92 |
|  | rs2338224 | A | 57.90 |
|  | rs10075801 | G | 64.79 |
|  | rs150616068 | A | 34.69 |
|  | rs2082382 | G | 99.11 |
|  | rs791357 | T | 104.77 |
|  | rs1334577 | A | 35.05 |
|  | rs1144700 | T | 53.86 |
|  | rs113977268 | A | 33.44 |
|  | rs2524079 | A | 301.76 |
|  | rs16895831 | T | 41.16 |
|  | rs9400271 | A | 85.95 |
|  | rs2451279 | A | 49.35 |
|  | rs2251188 | A | 34.08 |
|  | rs2158799 | C | 230.08 |
|  | rs56388170 | T | 354.85 |
|  | rs3735485 | A | 43.54 |
|  | rs8179 | T | 73.43 |
|  | rs445 | T | 260.89 |
|  | rs1991651 | C | 52.51 |
|  | rs12550612 | G | 41.35 |
|  | rs2979489 | G | 48.12 |
|  | rs7846314 | T | 196.26 |
|  | rs2436845 | A | 34.80 |
|  | rs2954031 | T | 36.19 |
|  | rs1982094 | T | 62.00 |
|  | rs6986779 | T | 59.93 |
|  | rs385893 | T | 52.74 |
|  | rs635634 | T | 78.40 |
|  | rs71191701 | C | 65.51 |
|  | rs12266014 | T | 140.48 |
|  | rs10995477 | C | 99.34 |
|  | rs2246941 | A | 33.55 |
|  | rs14408 | C | 193.38 |
|  | rs61897795 | G | 47.25 |
|  | rs192022 | G | 36.21 |
|  | rs238914 | A | 51.00 |
|  | rs8705 | A | 35.31 |
|  | rs111930700 | G | 45.18 |
|  | rs4761234 | C | 41.30 |
|  | rs11104881 | T | 41.72 |
|  | rs3184504 | T | 48.06 |
|  | rs1570884 | A | 36.62 |
|  | rs2038700 | C | 92.66 |
|  | rs11625487 | G | 48.45 |
|  | rs72699866 | A | 46.52 |
|  | rs12440045 | A | 45.47 |
|  | rs12101888 | T | 70.80 |
|  | rs60606273 | A | 40.13 |
|  | rs7168592 | T | 52.23 |
|  | rs6500550 | T | 38.51 |
|  | rs9926664 | G | 64.41 |
|  | rs8068017 | T | 36.08 |
|  | rs12600856 | C | 1092.23 |
|  | rs56378716 | G | 76.83 |
|  | rs11654074 | A | 72.88 |
|  | rs749780 | C | 34.16 |
|  | rs16978075 | C | 41.78 |
|  | rs1025688 | A | 39.00 |
|  | rs4147915 | A | 50.88 |
|  | rs4760 | G | 306.26 |
|  | rs73036517 | G | 139.30 |
|  | rs1800961 | T | 47.40 |
|  | rs9977672 | A | 37.94 |
|  | rs5013026 | C | 33.20 |
|  | rs35955747 | A | 33.33 |
| MCP-1 | rs56212190 | C | 23.55 |
|  | rs6694978 | C | 19.67 |
|  | rs7517040 | G | 26.70 |
|  | rs12075 | G | 198.72 |
|  | rs139026844 | A | 21.02 |
|  | rs12073356 | G | 21.02 |
|  | rs111995966 | G | 21.94 |
|  | rs143815843 | G | 19.58 |
|  | rs66694737 | G | 19.57 |
|  | rs2228467 | C | 82.12 |
|  | rs75265958 | G | 20.63 |
|  | rs12493471 | C | 51.54 |
|  | rs138591554 | T | 91.78 |
|  | rs112313229 | G | 27.65 |
|  | rs115077075 | C | 16.92 |
|  | rs2712431 | C | 20.94 |
|  | rs113089229 | C | 20.00 |
|  | rs111884151 | C | 19.06 |
|  | rs7019112 | G | 20.37 |
|  | rs61499291 | C | 19.44 |
|  | rs117218154 | C | 19.84 |
|  | rs10744620 | C | 23.96 |
|  | rs200603758 | A | 19.75 |
|  | rs9317045 | C | 23.09 |
|  | rs10145849 | G | 21.72 |
|  | rs147958317 | C | 19.11 |
|  | rs7197349 | G | 22.08 |
|  | rs186543342 | C | 19.44 |
|  | rs146522229 | C | 25.78 |
|  | rs149470547 | C | 20.01 |
| TNFa | rs116434579 | G | 19.95 |
|  | rs72841564 | C | 20.02 |
|  | rs10511404 | G | 20.23 |
|  | rs115669577 | G | 24.57 |
|  | rs111332265 | G | 24.95 |
|  | rs79105320 | G | 22.60 |
|  | rs10814274 | C | 19.77 |
|  | rs10834997 | G | 23.36 |
|  | rs7256693 | C | 20.23 |
|  | rs8121916 | C | 22.07 |
|  | rs72490194 | C | 20.11 |
| BDNF | rs17008416 | G | 24.68 |
|  | rs1647219 | C | 22.62 |
|  | rs8262 | G | 22.48 |
|  | rs9640740 | G | 21.62 |
|  | rs1458893 | C | 21.50 |
|  | rs11767756 | T | 21.34 |
|  | rs11987923 | A | 20.52 |
|  | rs7595430 | C | 20.29 |
|  | rs4484585 | C | 20.27 |
|  | rs221786 | C | 20.09 |
|  | rs12033122 | C | 19.63 |
|  | rs1891216 | T | 19.57 |
|  | rs10232201 | A | 19.53 |
|  | rs253414 | T | 19.47 |
|  | rs4595937 | G | 19.36 |
|  | rs12443771 | G | 19.29 |
|  | rs10244781 | T | 19.10 |
|  | rs11073742 | T | 19.09 |
|  | rs12149946 | G | 19.07 |
|  | rs4326076 | A | 18.81 |
|  | rs2185769 | A | 18.71 |
|  | rs2597348 | T | 18.59 |
|  | rs8121037 | T | 18.37 |
|  | rs12754916 | A | 18.36 |
|  | rs12425883 | G | 18.29 |
|  | rs1492970 | T | 18.13 |
|  | rs2894592 | T | 18.12 |
|  | rs8102487 | A | 17.97 |
|  | rs6902578 | C | 17.95 |
|  | rs17604707 | A | 17.86 |
|  | rs6736556 | T | 17.84 |
|  | rs2737645 | T | 17.75 |
|  | rs9950139 | A | 17.73 |
|  | rs17625497 | G | 17.58 |
|  | rs1456348 | G | 17.45 |
|  | rs2332172 | G | 17.27 |
|  | rs830307 | C | 17.11 |
|  | rs6735027 | A | 17.10 |
|  | rs4935325 | T | 16.98 |
|  | rs10848096 | T | 16.90 |
|  | rs2026390 | G | 16.81 |
|  | rs440139 | C | 16.67 |
|  | rs11569523 | C | 16.68 |
|  | rs4952029 | G | 16.65 |
|  | rs3134107 | G | 16.65 |
|  | rs11610288 | A | 16.62 |
|  | rs1419492 | C | 16.59 |
|  | rs2144490 | G | 16.54 |
|  | rs10169929 | A | 16.47 |
|  | rs3935935 | T | 16.36 |
|  | rs17057624 | T | 16.25 |
|  | rs9322150 | A | 16.27 |
|  | rs17517988 | A | 16.24 |
|  | rs7932591 | C | 16.18 |
|  | rs7443032 | T | 16.13 |
|  | rs13047973 | G | 16.13 |
|  | rs12762462 | G | 16.03 |
|  | rs11733491 | T | 15.99 |
|  | rs8031871 | C | 15.98 |
|  | rs7241931 | C | 15.98 |
|  | rs10755611 | G | 15.93 |
|  | rs10082174 | C | 15.84 |
|  | rs7013955 | G | 15.83 |
|  | rs7255992 | A | 15.81 |
|  | rs11658026 | A | 15.76 |
|  | rs12493846 | G | 15.66 |
|  | rs374433 | G | 15.63 |
|  | rs17021268 | G | 15.57 |
|  | rs11822374 | T | 15.53 |
|  | rs4900700 | A | 15.47 |
|  | rs11707806 | C | 15.44 |
|  | rs242888 | G | 15.38 |
|  | rs7936391 | G | 15.34 |
|  | rs9554751 | G | 15.34 |
|  | rs7716788 | G | 15.24 |
|  | rs4806255 | C | 15.20 |
|  | rs3806136 | T | 15.14 |
| CRP | rs12141273 | A | 20.57 |
|  | rs4133213 | A | 362.77 |
|  | rs3026932 | A | 27.73 |
|  | rs34217855 | T | 26.69 |
|  | rs7528267 | A | 56.85 |
|  | rs139779660 | T | 82.94 |
|  | rs1811472 | C | 992.98 |
|  | rs10753837 | T | 20.54 |
|  | rs12754747 | T | 26.06 |
|  | rs12132412 | A | 19.88 |
|  | rs7515963 | T | 20.63 |
|  | rs67090117 | T | 103.36 |
|  | rs61070846 | A | 24.43 |
|  | rs75460349 | A | 38.73 |
|  | rs61779359 | T | 56.56 |
|  | rs4916047 | A | 26.94 |
|  | rs2376015 | A | 601.36 |
|  | rs6695390 | T | 25.23 |
|  | rs78511209 | C | 27.70 |
|  | rs469773 | T | 44.18 |
|  | rs1115282 | T | 35.71 |
|  | rs6734238 | A | 124.24 |
|  | rs10210747 | A | 20.15 |
|  | rs62171052 | A | 24.46 |
|  | rs2682865 | A | 22.76 |
|  | rs12471471 | A | 25.24 |
|  | rs1877715 | A | 20.99 |
|  | rs67575134 | A | 21.09 |
|  | rs1260326 | T | 271.46 |
|  | rs1509394 | T | 27.56 |
|  | rs114839364 | T | 20.63 |
|  | rs62105327 | A | 35.12 |
|  | rs10167391 | A | 20.57 |
|  | rs12637835 | A | 24.06 |
|  | rs645040 | T | 20.79 |
|  | rs139261073 | A | 22.90 |
|  | rs113425220 | T | 23.75 |
|  | rs7356034 | A | 35.47 |
|  | rs139343479 | A | 21.26 |
|  | rs6792725 | A | 19.95 |
|  | rs10049413 | A | 39.27 |
|  | rs17050395 | A | 19.83 |
|  | rs10008492 | T | 27.38 |
|  | rs173816 | C | 20.62 |
|  | rs10066923 | A | 19.78 |
|  | rs34471628 | A | 43.76 |
|  | rs115823059 | A | 21.23 |
|  | rs870893 | A | 22.18 |
|  | rs1644005 | T | 20.15 |
|  | rs4354188 | T | 27.44 |
|  | rs1490384 | T | 40.21 |
|  | rs6903358 | A | 19.95 |
|  | rs56041438 | T | 20.02 |
|  | rs1474296 | C | 21.03 |
|  | rs59677899 | T | 25.21 |
|  | rs3130054 | T | 25.00 |
|  | rs2647062 | A | 37.64 |
|  | rs12197446 | A | 20.46 |
|  | rs118071618 | T | 21.60 |
|  | rs79614895 | A | 21.53 |
|  | rs11768827 | A | 25.41 |
|  | rs12154239 | T | 19.52 |
|  | rs10230276 | T | 22.60 |
|  | rs6464074 | A | 28.79 |
|  | rs13241897 | A | 55.18 |
|  | rs12673996 | T | 32.26 |
|  | rs2710804 | T | 22.45 |
|  | rs7797566 | A | 60.79 |
|  | rs10278040 | A | 24.77 |
|  | rs7008088 | A | 21.36 |
|  | rs6987444 | A | 66.69 |
|  | rs10956251 | T | 35.63 |
|  | rs2436135 | T | 20.14 |
|  | rs1545536 | T | 28.64 |
|  | rs7846476 | T | 20.04 |
|  | rs73174854 | T | 20.64 |
|  | rs7012637 | A | 158.88 |
|  | rs2482419 | T | 24.15 |
|  | rs2265688 | A | 22.11 |
|  | rs55969440 | T | 20.10 |
|  | rs505922 | T | 37.41 |
|  | rs74462579 | T | 21.12 |
|  | rs72825918 | T | 21.83 |
|  | rs10828264 | A | 23.50 |
|  | rs144621810 | D | 19.84 |
|  | rs114507486 | T | 22.16 |
|  | rs1332327 | T | 23.95 |
|  | rs12248932 | C | 24.51 |
|  | rs59255488 | T | 22.21 |
|  | rs10832027 | A | 40.31 |
|  | rs11031006 | A | 23.20 |
|  | rs4752829 | A | 32.94 |
|  | rs1582763 | A | 37.69 |
|  | rs7127808 | A | 20.01 |
|  | rs4584635 | A | 36.29 |
|  | rs7296380 | T | 37.99 |
|  | rs11065365 | A | 940.92 |
|  | rs2686343 | A | 42.09 |
|  | rs11047224 | C | 25.86 |
|  | rs147546981 | A | 20.21 |
|  | rs9738365 | A | 20.73 |
|  | rs76574446 | T | 22.11 |
|  | rs767455 | T | 28.86 |
|  | rs12813389 | A | 64.39 |
|  | rs17463269 | C | 22.62 |
|  | rs11620783 | T | 19.96 |
|  | rs2239222 | A | 74.19 |
|  | rs10459542 | T | 28.95 |
|  | rs9323608 | T | 23.12 |
|  | rs112635299 | T | 40.26 |
|  | rs1189402 | A | 35.43 |
|  | rs340005 | A | 71.27 |
|  | rs66481422 | A | 20.65 |
|  | rs12927172 | A | 24.76 |
|  | rs117986012 | A | 84.58 |
|  | rs2110840 | A | 61.05 |
|  | rs8057507 | T | 26.94 |
|  | rs8047395 | A | 37.73 |
|  | rs2550439 | T | 20.85 |
|  | rs7219250 | C | 21.73 |
|  | rs112238900 | T | 23.92 |
|  | rs3809758 | T | 20.51 |
|  | rs8073823 | T | 28.70 |
|  | rs16946790 | A | 21.84 |
|  | rs2384955 | T | 42.36 |
|  | rs2542160 | C | 36.00 |
|  | rs4092465 | A | 28.14 |
|  | rs7252707 | A | 20.15 |
|  | rs117690090 | T | 19.80 |
|  | rs62117205 | T | 28.90 |
|  | rs3810143 | T | 81.00 |
|  | rs6857 | T | 987.18 |
|  | rs204907 | A | 21.78 |
|  | rs6115099 | A | 28.53 |
|  | rs1800961 | T | 62.73 |
|  | rs6026470 | A | 21.85 |
|  | rs6027138 | A | 20.68 |
|  | rs6062509 | T | 23.47 |
|  | rs112646542 | T | 20.98 |
|  | rs4817983 | C | 68.15 |
|  | rs4821816 | A | 39.35 |
|  | rs9611441 | C | 27.19 |

^a^Approximated F-statistic (Pierce et al., 2011) representing instrument strength calculated using the formula: beta^2^ / standard error^2^.

| **Immune-Regulatory Protein** | **SNP** | **Effect Allele** | **F-Statistic^c^** |
| --- | --- | --- | --- |
| IL-1RA | rs6743376 | A | 196.00 |
|  | rs1542176 | C | 27.00 |
| sIL-2Rα | rs12722497 | C | 167.61 |
| IL-6^a^ – (Swerdlow et al., 2012) | rs12740969 | G | 36.00 |
|  | rs7529229 | C | 51.36 |
|  | rs4845371 | C | 22.75 |
| IL-6^a^ – (Sarwar et al., 2012) | rs2228145 | C | 397.03 |
| IL-6^b^ – (Georgakis et al., 2020) | rs73026617 | T | 48.59 |
|  | rs12083537 | A | 147.19 |
|  | rs4556348 | T | 65.20 |
|  | rs2228145 | A | 458.16 |
|  | rs11264224 | A | 66.55 |
|  | rs12059682 | T | 81.00 |
|  | rs34693607 | C | 41.68 |
| IL-16 | rs4778636 | G | 131.98 |
| IL-18 | rs1834481 | C | 144.00 |
| CRP | rs1205 | C | 1829.05 |
|  | rs3093077 | C | 658.98 |
|  | rs1130864 | A | 1000.86 |
|  | rs1800947 | C | 905.46 |
| BDNF | rs11030102 | C | 9.88 |

**Supplementary Table 2: Genetic variants included in *cis* analyses**

^a^These genetic instruments for IL-6 were obtained from Swerdlow et al (Swerdlow et al., 2012) and Sarwar et al (Sarwar et al., 2012), and are based on SNPs in the IL-6R region. These variants are associated with increased circulating IL-6 levels.

^b^This genetic instrument for IL-6 was obtained from Georgakis et al (Georgakis et al., 2020), which is also based on SNPs in the IL-6R region. However, Georgakis et al coded their data to reflect associations of these variants with increased circulating CRP levels. We used the same coding for our analysis.

^c^Approximated F-statistic (Pierce et al., 2011) representing instrument strength calculated using the formula: beta^2^ / standard error^2^.

**Supplementary Figure 1: Illustration of Instruments Used for IL-6**


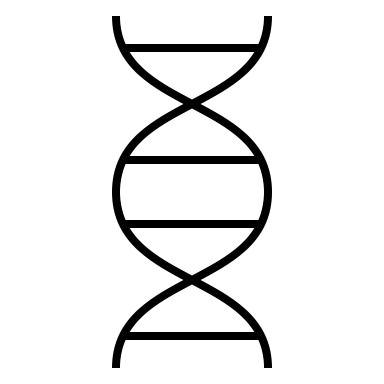


*IL6R* gene


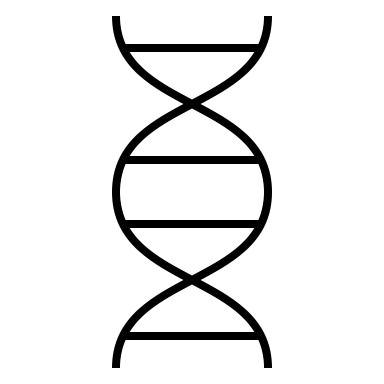

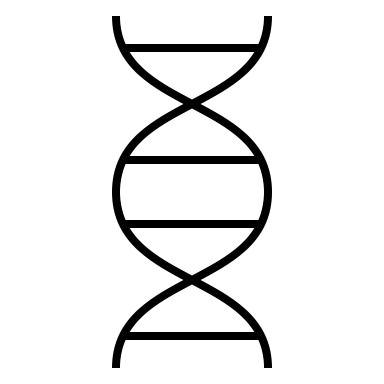

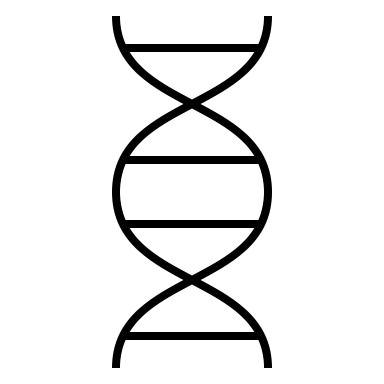


Swerdlow *et al* instrument and effect allele

rs12740969 - G

rs7529229 - C

rs4845371 - C

Sarwar *et al* instrument and effect allele

rs2228145 - C

Georgakis *et al* instrument and effect allele

rs2228145 - A

rs73026617 - T

rs12083537 - A

rs4556348 - T

rs11264224 - A

rs12059682 - T

rs34693607 - C

SNP Effects on IL-6 Levels

SNP Effects on CRP Levels

All three IL-6 instruments are based on variants in the *IL6R* region. While variants in the Swerdlow *et al* and Sarwar *et al* instruments increase circulating IL-6 levels, the Sarwar *et al* instrument in particular (*IL-6R* Asp358Ala; rs2228145 A>C) has been shown in a peripheral blood mononuclear cell-based experiment (Ferreira et al., 2013) to decrease CRP levels in carriers of the minor 358Ala allele (effect allele C) as a result of impaired IL-6 classical signalling, due to decreased expression of membrane-bound IL-6R (Ferreira et al., 2013). This variant is in high LD with the variants used in the Swerdlow *et al* instrument. Therefore, based on the existing literature, variants used in the Swerdlow *et al* and Sarwar *et al* instruments increase circulating IL-6 levels, yet downregulate IL-6 classical signalling, leading to decreased CRP levels. The Georgakis *et al* instrument coded SNPs to reflect higher circulating CRP levels, as a potential indicator of upregulated IL-6 signalling (Georgakis et al., 2020). For example, the *IL-6R* variant rs2228145 features in both the Sarwar *et al* and Georgakis *et al*  IL-6 instruments. However, the Sarwar *et al* instrument used the C allele as the effect allele (associated with lower CRP due to downregulated IL-6 classical signalling), but the Georgakis *et al* instrument used the A allele as the effect allele (associated with higher CRP levels due to upregulated IL-6 classical signalling).

**Supplementary Table 3: Median and Range of F-Statistics for SNPs Included in *Trans* Analyses**

| **Immune-Regulatory Protein** | **Median F-Statistic** | **Range of F-Statistics** |
| --- | --- | --- |
| IL-1RA | 22.22 | 19.49-26.51 |
| sIL-2Rα | 21.03 | 19.51-167.61 |
| IL-4 | 21.70 | 19.71-26.63 |
| IL-5 | 20.91 | 19.40-37.93 |
| IL-7 | 22.14 | 19.37-169.84 |
| IL-8 | 20.44 | 19.57-25.95 |
| IL-9 | 20.60 | 19.53-26.46 |
| IL-10 | 22.44 | 19.49-298.98 |
| IL-12 | 21.40 | 19.64-564.29 |
| IL-13 | 21.14 | 19.60-292.85 |
| IL-16 | 24.42 | 20.16-131.98 |
| IL-17 | 21.35 | 19.51-38.97 |
| IL-18 | 22.97 | 19.73-96.17 |
| CRP | 24.96 | 19.52-992.98 |
| TNFa | 20.23 | 19.77-24.95 |
| MCP-1 | 20.98 | 16.92-198.72 |
| BDNF | 16.98 | 15.14-24.68 |
| Neutrophils | 50.39 | 33.20-1092.23 |
| Lymphocytes | 59.87 | 33.21-606.36 |

**Supplementary Table 4: Median and Range of F-Statistics for SNPs Included in *Cis* Analyses**

| **Immune-Regulatory Protein** | **Median F-Statistic** | **Range of F-Statistics** |
| --- | --- | --- |
| IL-1RA | 111.50 | 27.00-196.00 |
| sIL-2Rα | 167.61^a^ | 167.61^a^ |
| IL-6 –(Swerdlow et al., 2012) | 36.00 | 22.75-51.36 |
| IL-6 – (Sarwar et al., 2012) | 397.03^a^ | 397.03^a^ |
| IL-6 – (Georgakis et al., 2020) | 66.66 | 41.68-458.16 |
| IL-16 | 131.98^a^ | 131.98^a^ |
| IL-18 | 144.00^a^ | 144.00^a^ |
| CRP | 953.16 | 658.98-1829.05 |
| BDNF | 9.88^a^ | 9.88^a^ |

^a^Single variant used as instrument

**Supplementary Methods**

**Definitions and Samples for Psychiatric Outcomes**

**Schizophrenia**

The summary statistics for schizophrenia were derived from a GWAS meta-analysis (Pardinas et al., 2018) of GWAS data from the Psychiatric Genomics Consortium (PGC) 2 dataset (Schizophrenia Working Group of the Psychiatric Genomics, 2014) and data from a mandatory clozapine blood-monitoring system of treatment-resistant schizophrenia patients who were prescribed clozapine. A clinical diagnosis meeting the consensus research DSM-IV diagnosis of schizophrenia was validated with a research interview for consenting participants.

**Depression**

The summary statistics for depression were derived from a GWAS meta-analysis (Wray et al., 2018) of seven European and North American cohorts with genotype data available. We used data from five cohorts where participants were evaluated directly and met the DSM-IV or ICD-10 criteria for major depressive disorder (MDD). Different methods were used to classify MDD case status amongst the included cohorts, and these included structured diagnostic interviews or diagnoses from national inpatient electronic records.

**Bipolar Disorder**

The summary statistics for bipolar disorder were derived from a GWAS meta-analysis (Mullins et al., 2021) of 57 bipolar disorder cohorts from Europe, North America and Canada. In each cohort, lifetime bipolar disorder case status was ascertained either through direct clinical interview meeting either DSM-IV, ICD-9 or ICD-10 criteria, hospital records or ICD-code searches.

**MR Analysis Methods**

**Inverse Weighted Variance (IVW) Analysis**

IVW consists of a weighted linear regression of SNP-exposure SNP-outcome effect estimates. The IVW estimate is the inverse variance weighted mean of ratio estimates from 2 or more instruments (Burgess et al., 2013), and assumes that all SNPs are valid instruments or that the sum of directional bias is zero. Since the intercept is an estimate of average pleiotropic effects across IVs, in an IVW approach the intercept is fixed to 0.

**Weighted Median Analysis**

The weighted median is the median of the weighted empirical distribution function of individual SNP ratio estimates. This method provides a consistent effect estimate if more than 50% of the information comes from valid SNPs (Bowden et al., 2016).

**MR Egger Analysis**

MR-Egger regression consists of a weighted linear regression similar to IVW, with the assumption that horizontal pleiotropic effects and SNP-exposure associations are uncorrelated (i.e. the InSIDE assumption is not violated (Bowden et al., 2015)), therefore the intercept is not fixed. MR Egger regression provides a valid effect estimate even if all SNPs are invalid instruments but assumes that uncertainty in the SNP-exposure association estimates is negligible (the ‘NOME’ assumption (Bowden et al., 2017)).

**Mendelian Randomization Pleiotropy Residual Sum and Outlier (MR-PRESSO) Test**

MR-PRESSO (Verbanck et al., 2018) relies on a regression framework where the variants’ effects on the outcome are regressed on the same variants’ effects on exposure, with the slope of the regression line providing an estimate of the causal effect of the exposure on the outcome. The MR-PRESSO global test evaluates overall horizontal pleiotropy amongst all IVs in a single MR test by comparing the observed distance of all the variants to the regression line (residual sum of squares) to the expected distance under the null hypothesis of no horizontal pleiotropy. The MR-PRESSO outlier test evaluates the presence of specific horizontal pleiotropic outlier variants by using the observed and expected distributions of the tested variant. Finally, the MR-PRESSO distortion test evaluates the significance of the distortion between the causal estimate before and after removal of the horizontal pleiotropic outlier variants (detected from the outlier test of MR-PRESSO).

**Supplementary Table 5: Power Calculations for Two Sample Mendelian Randomization**

| **Immune-Regulatory Protein** | **Variance Explained (R^2^) by the Instrument** | **Minimum Causal Effect (Odds Ratio) Detectable With 80% Power** | | |
| --- | --- | --- | --- | --- |
|  |  | **Schizophrenia** | **Major Depression** | **Bipolar Disorder** |
| IL-1RA, sIL-2Rα, IL-4, IL-5, IL-7, IL-8, IL-9, IL-10, IL-12, IL-13, IL-16, IL-17, IL-18, TNFa, MCP-1(Ahola-Olli et al., 2017)^a^ | 0.010  0.025  0.050  0.075  0.100 | 0.83-1.20  0.89-1.12  0.92-1.09  0.93-1.07  0.94-1.06 | 0.91-1.10  0.94-1.06  0.96-1.05  0.96-1.04  0.97-1.03 | 0.86-1.16  0.91-1.10  0.93-1.07  0.94-1.06  0.95-1.05 |
| IL-6 (Swerdlow et al., 2012) | 0.011 | 0.84-1.19 | 0.91-1.09 | 0.87-1.15 |
| IL-6 (Collaboration et al., 2012) | 0.010 | 0.83-1.20 | 0.91-1.10 | 0.86-1.16 |
| IL-6 (Georgakis et al., 2020) | 0.008 | 0.82-1.22 | 0.90-1.11 | 0.85-1.18 |
| CRP (Ligthart et al., 2018) | 0.065 | 0.93-1.08 | 0.96-1.04 | 0.94-1.06 |
| Neutrophils (Astle et al., 2016) | 0.080 | 0.93-1.07 | 0.96-1.04 | 0.95-1.06 |
| Lymphocytes (Astle et al., 2016) | 0.100 | 0.94-1.06 | 0.97-1.03 | 0.95-1.05 |

^a^R^2^/genetic association of the instrument not reported, so range of possible R^2^ values are shown

Note: Power calculations were conducted using Burgess’ online calculator (<http://sb452.shinyapps.io>/power) (Burgess, 2014). Where possible, R^2^ was taken from the original GWAS. Where this was not reported, R^2^ was approximated using the formula 2*beta_X^2*MAF* (1-MAF), where beta_X is the genetic association with the instrument (in SDs). Causal effect refers to the log odds in SD units converted to odds ratio per SD change in exposure.

**Supplementary Table 6: ORs (95% CIs) for MR Associations between Immune-Regulatory Proteins (*Trans* Variants) and Schizophrenia**

| **Outcome / Exposure** | **Method** | **Odds Ratio (95% C.I.)** | **P-value** |
| --- | --- | --- | --- |
| IL-1RA | IVW | 0.99 (0.92-1.07) | 0.806 |
|  | Weighted Median | 1.01 (0.94-1.09) | 0.734 |
|  | MR Egger | 0.93 (0.74-1.17) | 0.569 |
| sIL-2Rα | IVW | 1.03 (0.98-1.07) | 0.254 |
|  | Weighted Median | 1.04 (1.00-1.09) | 0.080 |
|  | MR Egger | 1.01 (0.94-1.09) | 0.774 |
| IL-4 | IVW | 0.99 (0.91-1.08) | 0.821 |
|  | Weighted Median | 1.00 (0.91-1.10) | 0.930 |
|  | MR Egger | 1.01 (0.87-1.17) | 0.900 |
| IL-5 | IVW | 1.02 (0.96-1.09) | 0.513 |
|  | Weighted Median | 1.01 (0.94-1.08) | 0.778 |
|  | MR Egger | 1.08 (0.93-1.25) | 0.342 |
| IL-7 | IVW | 1.01 (0.98-1.05) | 0.524 |
|  | Weighted Median | 1.01 (0.96-1.06) | 0.813 |
|  | MR Egger | 1.02 (0.94-1.09) | 0.693 |
| IL-8 | IVW | 1.01 (0.97-1.05) | 0.783 |
|  | Weighted Median | 1.03 (0.98-1.09) | 0.244 |
|  | MR Egger | 1.01 (0.94-1.09) | 0.769 |
| IL-9 | IVW | 1.06 (1.01-1.11) | 0.011* |
|  | Weighted Median | 1.05 (1.01-1.11) | 0.143 |
|  | MR Egger | 1.02 (0.91-1.13) | 0.772 |
| IL-10 | IVW | 0.98 (0.93-1.03) | 0.397 |
|  | Weighted Median | 0.99 (0.93-1.06) | 0.770 |
|  | MR Egger | 1.03 (0.93-1.14) | 0.575 |
| IL-12 | IVW | 0.99 (0.96-1.03) | 0.707 |
|  | Weighted Median | 0.99 (0.95-1.04) | 0.787 |
|  | MR Egger | 1.00 (0.94-1.06) | 0.921 |
| IL-13 | IVW | 1.02 (0.98-1.06) | 0.283 |
|  | Weighted Median | 1.00 (0.96-1.04) | 0.901 |
|  | MR Egger | 1.04 (0.97-1.12) | 0.299 |
| IL-16 | IVW | 0.99 (0.96-1.02) | 0.532 |
|  | Weighted Median | 0.99 (0.95-1.03) | 0.637 |
|  | MR Egger | 0.98 (0.93-1.04) | 0.544 |
| IL-17 | IVW | 0.98 (0.92-1.04) | 0.454 |
|  | Weighted Median | 0.98 (0.89-1.08) | 0.729 |
|  | MR Egger | 0.94 (0.81-1.09) | 0.454 |
| IL-18 | IVW | 1.01 (0.98-1.04) | 0.605 |
|  | Weighted Median | 0.99 (0.95-1.04) | 0.698 |
|  | MR Egger | 0.98 (0.91-1.04) | 0.480 |
| CRP | IVW | 0.92 (0.85-0.99) | 0.036* |
|  | Weighted Median | 0.85 (0.77-0.93) | 0.004* |
|  | MR Egger | 0.96 (0.84-1.09) | 0.562 |
| TNFa | IVW | 1.03 (0.99-1.09) | 0.143 |
|  | Weighted Median | 1.01 (0.94-1.08) | 0.799 |
|  | MR Egger | 1.06 (0.96-1.18) | 0.295 |
| MCP1 | IVW | 0.96 (0.91-0.99) | 0.049* |
|  | Weighted Median | 0.93 (0.87-0.97) | 0.016* |
|  | MR Egger | 0.93 (0.85-1.03) | 0.185 |
| BDNF | IVW | 1.00 (0.99-1.01) | 0.948 |
|  | Weighted Median | 1.01 (1.00-1.02) | 0.119 |
|  | MR Egger | 1.00 (0.99-1.01) | 0.924 |
| Neutrophils | IVW | 1.04 (0.94-1.14) | 0.497 |
|  | Weighted Median | 1.00 (0.89-1.13) | 0.950 |
|  | MR Egger | 1.00 (0.83-1.22) | 0.976 |
| Lymphocytes | IVW | 1.00 (0.90-1.11) | 0.977 |
|  | Weighted Median | 0.95 (0.84-1.09) | 0.472 |
|  | MR Egger | 1.02 (0.75-1.37) | 0.922 |

**p*<0.05 ^†^*p*<0.003 (Bonferroni corrected for 19 exposures)

**Supplementary Table 7: ORs (95% CIs) for MR Associations between Immune-Regulatory Proteins (*Trans* Variants) and Bipolar Disorder**

| **Exposure** | **Method** | **Odds Ratio (95% C.I.)** | **P-value** |
| --- | --- | --- | --- |
| IL-1RA | IVW | 1.00 (0.88-1.14) | 0.989 |
|  | Weighted Median | 1.02 (0.95-1.09) | 0.567 |
|  | MR Egger | 1.36 (0.98-1.88) | 0.095 |
| sIL-2Rα | IVW | 1.01 (0.97-1.05) | 0.666 |
|  | Weighted Median | 1.02 (0.98-1.07) | 0.305 |
|  | MR Egger | 1.01 (0.94-1.09) | 0.714 |
| IL-4 | IVW | 0.98 (0.92-1.04) | 0.485 |
|  | Weighted Median | 0.98 (0.91-1.07) | 0.686 |
|  | MR Egger | 0.94 (0.85-1.04) | 0.257 |
| IL-5 | IVW | 1.00 (0.95-1.05) | 0.940 |
|  | Weighted Median | 0.99 (0.94-1.05) | 0.829 |
|  | MR Egger | 0.96 (0.85-1.09) | 0.524 |
| IL-7 | IVW | 0.99 (0.96-1.03) | 0.677 |
|  | Weighted Median | 1.01 (0.96-1.06) | 0.718 |
|  | MR Egger | 0.98 (0.91-1.05) | 0.522 |
| IL-8 | IVW | 0.98 (0.94-1.03) | 0.494 |
|  | Weighted Median | 0.97 (0.92-1.03) | 0.346 |
|  | MR Egger | 0.99 (0.92-1.08) | 0.897 |
| IL-9 | IVW | 0.98 (0.92-1.05) | 0.604 |
|  | Weighted Median | 0.95 (0.89-1.02) | 0.130 |
|  | MR Egger | 1.00 (0.95-1.17) | 0.996 |
| IL-10 | IVW | 1.02 (0.96-1.08) | 0.592 |
|  | Weighted Median | 1.00 (0.94-1.07) | 0.909 |
|  | MR Egger | 1.01 (0.89-1.15) | 0.833 |
| IL-12 | IVW | 1.01 (0.95-1.06) | 0.855 |
|  | Weighted Median | 1.01 (0.96-1.05) | 0.808 |
|  | MR Egger | 1.02 (0.93-1.12) | 0.674 |
| IL-13 | IVW | 0.99 (0.94-1.03) | 0.630 |
|  | Weighted Median | 1.00 (0.96-1.04) | 0.934 |
|  | MR Egger | 1.03 (0.95-1.11) | 0.472 |
| IL-16 | IVW | 1.00 (0.95-1.04) | 0.839 |
|  | Weighted Median | 1.03 (0.98-1.08) | 0.192 |
|  | MR Egger | 1.03 (0.96-1.11) | 0.406 |
| IL-17 | IVW | 1.02 (0.95-1.09) | 0.642 |
|  | Weighted Median | 1.01 (0.92-1.11) | 0.824 |
|  | MR Egger | 0.90 (0.78-1.05) | 0.212 |
| IL-18 | IVW | 0.99 (0.96-1.02) | 0.461 |
|  | Weighted Median | 0.99 (0.95-1.04) | 0.703 |
|  | MR Egger | 0.98 (0.92-1.04) | 0.527 |
| CRP | IVW | 0.98 (0.90-1.06) | 0.548 |
|  | Weighted Median | 1.00 (0.90-1.10) | 0.968 |
|  | MR Egger | 0.99 (0.86-1.13) | 0.828 |
| TNFa | IVW | 0.98 (0.94-1.03) | 0.417 |
|  | Weighted Median | 0.95 (0.89-1.02) | 0.176 |
|  | MR Egger | 0.92 (0.83-1.02) | 0.149 |
| MCP1 | IVW | 1.02 (0.97-1.07) | 0.534 |
|  | Weighted Median | 0.98 (0.92-1.04) | 0.447 |
|  | MR Egger | 0.96 (0.86-1.06) | 0.401 |
| BDNF | IVW | 1.00 (0.99-1.01) | 0.785 |
|  | Weighted Median | 1.00 (0.00-1.01) | 0.770 |
|  | MR Egger | 0.99 (0.98-1.01) | 0.947 |
| Neutrophils | IVW | 0.96 (0.86-1.07) | 0.430 |
|  | Weighted Median | 0.94 (0.82-1.07) | 0.329 |
|  | MR Egger | 1.08 (0.82-1.34) | 0.467 |
| Lymphocytes | IVW | 0.95 (0.87-1.04) | 0.301 |
|  | Weighted Median | 0.98 (0.87-1.10) | 0.694 |
|  | MR Egger | 1.12 (0.88-1.43) | 0.362 |

**p*<0.05 ^†^*p*<0.003 (Bonferroni corrected for 19 exposures)

**Supplementary Table 8: ORs (95% CIs) for MR Associations between Immune-Regulatory Proteins (*Trans* Variants) and Major Depression**

| **Exposure** | **Method** | **Odds Ratio (95% C.I.)** | **P-value** |
| --- | --- | --- | --- |
| IL-1RA | IVW | 1.01 (0.97-1.05) | 0.629 |
|  | Weighted Median | 1.02 (0.97-1.08) | 0.382 |
|  | MR Egger | 1.01 (0.89-1.14) | 0.860 |
| sIL-2Rα | IVW | 1.01 (0.98-1.04) | 0.382 |
|  | Weighted Median | 1.01 (0.97-1.05) | 0.650 |
|  | MR Egger | 1.02 (0.98-1.07) | 0.318 |
| IL-4 | IVW | 1.00 (0.95-1.06) | 0.964 |
|  | Weighted Median | 1.00 (0.93-1.07) | 0.946 |
|  | MR Egger | 1.00 (0.91-1.09) | 0.922 |
| IL-5 | IVW | 0.98 (0.95-1.02) | 0.295 |
|  | Weighted Median | 0.98 (0.93-1.03) | 0.400 |
|  | MR Egger | 0.98 (0.90-1.06) | 0.563 |
| IL-7 | IVW | 0.99 (0.96-1.02) | 0.559 |
|  | Weighted Median | 0.99 (0.96-1.03) | 0.728 |
|  | MR Egger | 1.01 (0.96-1.07) | 0.720 |
| IL-8 | IVW | 1.01 (0.97-1.04) | 0.741 |
|  | Weighted Median | 1.00 (0.97-1.04) | 0.813 |
|  | MR Egger | 1.01 (0.95-1.05) | 0.961 |
| IL-9 | IVW | 1.01 (0.97-1.04) | 0.651 |
|  | Weighted Median | 1.01 (0.97-1.06) | 0.629 |
|  | MR Egger | 1.01 (0.93-1.10) | 0.833 |
| IL-10 | IVW | 1.00 (0.96-1.04) | 0.883 |
|  | Weighted Median | 1.00 (0.95-1.05) | 0.983 |
|  | MR Egger | 0.99 (0.92-1.08) | 0.964 |
| IL-12 | IVW | 1.00 (0.97-1.03) | 0.921 |
|  | Weighted Median | 1.00 (0.96-1.04) | 0.980 |
|  | MR Egger | 1.00 (0.95-1.06) | 0.921 |
| IL-13 | IVW | 1.00 (0.98-1.03) | 0.955 |
|  | Weighted Median | 1.00 (0.97-1.04) | 0.911 |
|  | MR Egger | 1.01 (0.97-1.06) | 0.624 |
| IL-16 | IVW | 1.01 (0.98-1.03) | 0.604 |
|  | Weighted Median | 1.00 (0.97-1.05) | 0.802 |
|  | MR Egger | 1.00 (0.96-1.05) | 0.834 |
| IL-17 | IVW | 1.00 (0.95-1.05) | 0.979 |
|  | Weighted Median | 0.99 (0.92-1.06) | 0.800 |
|  | MR Egger | 1.00 (0.89-1.13) | 0.987 |
| IL-18 | IVW | 1.00 (0.98-1.03) | 0.856 |
|  | Weighted Median | 1.01 (0.97-1.04) | 0.717 |
|  | MR Egger | 1.00 (0.95-1.05) | 0.932 |
| CRP | IVW | 1.01 (0.98-1.07) | 0.355 |
|  | Weighted Median | 1.02 (0.95-1.09) | 0.604 |
|  | MR Egger | 1.03 (0.95-1.11) | 0.521 |
| TNFa | IVW | 1.01 (0.97-1.05) | 0.606 |
|  | Weighted Median | 1.01 (0.96-1.05) | 0.725 |
|  | MR Egger | 0.99 (0.93-1.07) | 0.883 |
| MCP1 | IVW | 1.00 (0.97-1.04) | 0.892 |
|  | Weighted Median | 1.01 (0.96-1.06) | 0.679 |
|  | MR Egger | 1.01 (0.94-1.09) | 0.745 |
| BDNF | IVW | 1.00 (0.99-1.01) | 0.703 |
|  | Weighted Median | 0.99 (0.98-1.01) | 0.950 |
|  | MR Egger | 1.00 (0.99-1.01) | 0.907 |
| Neutrophils | IVW | 1.02 (0.96-1.08) | 0.529 |
|  | Weighted Median | 1.04 (0.95-1.14) | 0.398 |
|  | MR Egger | 1.03 (0.91-1.16) | 0.660 |
| Lymphocytes | IVW | 1.00 (0.94-1.06) | 0.897 |
|  | Weighted Median | 1.03 (0.94-1.12) | 0.522 |
|  | MR Egger | 1.06 (0.89-1.27) | 0.484 |

**p*<0.05 ^†^*p*<0.003 (Bonferroni corrected for 19 exposures)

**Supplementary Table 9: ORs (95% CIs) for MR Associations between Immune-Regulatory Proteins (*Cis* Variants) and Schizophrenia, Bipolar Disorder and Major Depression**

| **Outcome / Exposure** | **Method** | **Odds Ratio (95% C.I.)** | **P-value** |
| --- | --- | --- | --- |
| **Schizophrenia** | | | |
| IL-1RA | IVW | 1.07 (0.88-1.30) | 0.497 |
| sIL-2Rα | Wald Ratio | 1.07 (1.01-1.12) | 0.017* |
| IL-6 (Swerdlow et al., 2012) | IVW | 1.24 (1.04-1.47) | 0.014* |
|  | Weighted Median | 1.17 (0.96-1.41) | 0.115 |
|  | MR Egger | 2.14 (0.49-9.46) | 0.498 |
| IL-6 (Sarwar et al., 2012) | Wald Ratio | 1.10 (1.03-1.17) | 0.005* |
| IL-6 (Georgakis et al., 2020) | IVW  Weighted Median  MR Egger | 0.72 (0.58-0.88)  0.74 (0.60-0.90)  0.85 (0.42-1.72) | 0.001^†^  0.002^†^  0.669 |
| IL-12 | IVW | 0.88 (0.74-1.06) | 0.173 |
| IL-16 | Wald Ratio | 0.99 (0.95-1.04) | 0.680 |
| IL-18 | Wald Ratio | 1.10 (0.91-1.32) | 0.314 |
| CRP | IVW | 0.93 (0.88-0.99) | 0.046* |
|  | Weighted Median | 0.92 (0.86-0.99) | 0.038* |
|  | MR Egger | 0.93 (0.81-1.08) | 0.420 |
| BDNF | Wald Ratio | 0.97 (0.94-1.00) | 0.052* |
| **Bipolar Disorder** | | | |
| IL-1RA | IVW | 0.98 (0.86-1.12) | 0.764 |
| sIL-2Rα | Wald Ratio | 1.03 (0.98-1.09) | 0.246 |
| IL-6 (Swerdlow et al., 2012) | IVW  Weighted Median  MR Egger | 1.02 (0.88-1.17) 1.02 (0.88-1.20) 0.97 (0.33-2.84) | 0.796 0.767 0.959 |
| IL-6 (Sarwar et al., 2012) | Wald Ratio | 1.01 (0.95-1.07) | 0.825 |
| IL-6 (Georgakis et al., 2020) | IVW  Weighted Median  MR Egger | 0.98 (0.76-1.27) 1.03 (0.86-1.23) 0.99 (0.36-2.76) | 0.892 0.746 0.986 |
| IL-12 | Wald Ratio | 1.02 (0.80-1.29) | 0.889 |
| IL-16 | Wald Ratio | 1.04 (0.99-1.09) | 0.112 |
| IL-18 | Wald Ratio | 1.08 (0.91-1.26) | 0.390 |
| CRP | IVW  Weighted Median  MR Egger | 1.01 (0.94-1.10) 1.01 (0.93-1.10) 1.06 (0.66-1.70) | 0.708 0.786 0.853 |
| BDNF | Wald Ratio | 0.97 (0.94-1.00) | 0.097 |
| **Major Depression** | | | |
| IL-1RA | IVW | 1.04 (0.93-1.17) | 0.510 |
| sIL-2Rα | Wald Ratio | 1.00 (0.96-1.04) | 0.827 |
| IL-6 (Swerdlow et al., 2012) | IVW  Weighted Median  MR Egger | 1.07 (0.95-1.21)  1.07 (0.94-1.23)  1.12 (0.45-2.77) | 0.243  0.311  0.848 |
| IL-6 (Sarwar et al., 2012) | Wald Ratio | 0.92 (0.77-1.10) | 0.350 |
| IL-6 (Georgakis et al., 2020) | IVW  Weighted Median  MR Egger | 0.95 (0.84-1.08)  0.94 (0.80-1.10)  0.87 (0.58-1.30) | 0.437  0.462  0.535 |
| IL-12 | Wald Ratio | 1.02 (0.83-1.26) | 0.852 |
| IL-16 | Wald Ratio | 1.00 (0.97-1.04) | 0.854 |
| IL-18 | Wald Ratio | 1.05 (0.90-1.22) | 0.535 |
| CRP | IVW  Weighted Median  MR Egger | 1.00 (0.94-1.05)  0.99 (0.93-1.06)  0.97 (0.77-1.21) | 0.907  0.839  0.794 |
| BDNF | Wald Ratio | 1.03 (0.99-1.04) | 0.344 |

**p*<0.05; ^†^*p*<0.005 (Bonferroni corrected for 10 exposures);

**Supplementary Table 10: ORs (95% CIs) for MVMR Associations of IL-6, sIL-2Rα and CRP (*Cis* Variants) with Schizophrenia, Bipolar Disorder and Depression**

| **Outcome** | **Exposure** | **IVW Odds Ratio (95% C.I.)** | **P-value** |
| --- | --- | --- | --- |
| Schizophrenia | IL-6 (Swerdlow et al., 2012) | 1.26 (1.05-1.52) | 0.013^†^ |
|  | CRP | 1.01 (0.88-1.15) | 0.927 |
|  | sIL-2Rα | 1.05 (1.01-1.10) | 0.047* |
| Major Depression | IL-6 (Swerdlow et al., 2012) | 1.08 (1.03-1.12) | <0.001^†^ |
|  | CRP | 1.01 (0.97-1.04) | 0.638 |
|  | sIL-2Rα | 1.00 (0.99-1.01) | 0.622 |
| Bipolar Disorder | IL-6 (Swerdlow et al., 2012) | 1.01 (0.95-1.08) | 0.733 |
|  | CRP | 0.98 (0.93-1.02) | 0.341 |
|  | sIL-2Rα | 0.97 (0.95-1.01) | 0.204 |

**p*<0.05; ^†^*p*<0.017 (Bonferroni corrected for 3 exposures)

**Supplementary Table 11: ORs (95% CIs) for Bidirectional MR Associations of Schizophrenia with Immune-Regulatory Proteins**

| **Outcome** | **Method** | **B (SE)** | **P-value** |
| --- | --- | --- | --- |
| IL-1RA | IVW | 0.07 (0.08) | 0.368 |
|  | Weighted Median | 0.06 (0.11) | 0.598 |
|  | MR Egger | 0.20 (0.39) | 0.607 |
| sIL-2Rα | IVW | 0.09 (0.08) | 0.208 |
|  | Weighted Median | 0.14 (0.11) | 0.179 |
|  | MR Egger | 0.48 (0.37) | 0.209 |
| IL-4 | IVW | 0.06 (0.07) | 0.374 |
|  | Weighted Median | 0.12 (0.09) | 0.208 |
|  | MR Egger | 0.06 (0.34) | 0.852 |
| IL-5 | IVW | 0.16 (0.10) | 0.094 |
|  | Weighted Median | 0.24 (0.14) | 0.087 |
|  | MR Egger | 0.80 (0.47) | 0.094 |
| IL-6 | IVW | 0.04 (0.05) | 0.482 |
|  | Weighted Median | 0.03 (0.07) | 0.662 |
|  | MR Egger | 0.08 (0.24) | 0.733 |
| IL-7 | IVW | 0.13 (0.11) | 0.239 |
|  | Weighted Median | 0.03 (0.15) | 0.825 |
|  | MR Egger | 0.16 (0.53) | 0.763 |
| IL-8 | IVW | 0.03 (0.09) | 0.741 |
|  | Weighted Median | -0.01 (0.13) | 0.913 |
|  | MR Egger | 0.36 (0.49) | 0.461 |
| IL-9 | IVW | -0.06 (0.10) | 0.501 |
|  | Weighted Median | 0.03 (0.13) | 0.809 |
|  | MR Egger | 0.11 (0.47) | 0.805 |
| IL-10 | IVW | -0.01 (0.07) | 0.575 |
|  | Weighted Median | 0.00 (0.09) | 0.975 |
|  | MR Egger | -0.19 (0.34) | 0.902 |
| IL-12 | IVW | 0.08 (0.07) | 0.102 |
|  | Weighted Median | 0.06 (0.07) | 0.400 |
|  | MR Egger | 0.22 (0.25) | 0.377 |
| IL-13 | IVW | 0.20 (0.18) | 0.154 |
|  | Weighted Median | 0.19 (0.14) | 0.187 |
|  | MR Egger | 0.22 (0.49) | 0.654 |
| IL-16 | IVW | -0.10 (0.10) | 0.291 |
|  | Weighted Median | -0.09 (0.14) | 0.517 |
|  | MR Egger | 0.73 (0.48) | 0.132 |
| IL-17 | IVW | 0.07 (0.05) | 0.163 |
|  | Weighted Median | 0.00 (0.08) | 0.970 |
|  | MR Egger | -0.02 (0.25) | 0.251 |
| IL-18 | IVW | -0.04 (0.09) | 0.699 |
|  | Weighted Median | -0.06 (0.13) | 0.626 |
|  | MR Egger | -0.54 (0.45) | 0.231 |
| CRP | IVW | 0.00 (0.06) | 0.938 |
|  | Weighted Median | 0.00 (0.08) | 1.000 |
|  | MR Egger | -0.01 (0.25) | 0.976 |
| MCP-1 | IVW | 0.02 (0.05) | 0.670 |
|  | Weighted Median | 0.09 (0.07) | 0.229 |
|  | MR Egger | -0.26 (0.24) | 0.284 |
| TNFa | IVW | 0.03 (0.08) | 0.689 |
|  | Weighted Median | 0.08 (0.12) | 0.494 |
|  | MR Egger | 0.48 (0.39) | 0.217 |
| Neutrophils | IVW | 0.04 (0.06) | 0.443 |
|  | Weighted Median | 0.08 (0.10) | 0.512 |
|  | MR Egger | 0.22 (0.19) | 0.532 |
| Lymphocytes | IVW | 0.09 (0.11) | 0.885 |
|  | Weighted Median | 0.07 (0.09) | 0.294 |
|  | MR Egger | 0.22 (0.31) | 0.776 |

**p*<0.05 ^†^*p*<0.003 (Bonferroni corrected for 19 exposures).

**Supplementary Table 12: ORs (95% CIs) for Bidirectional MR Associations of Bipolar Disorder with Immune-Regulatory Proteins**

| **Outcome** | **Method** | **B (SE)** | **P-value** |
| --- | --- | --- | --- |
| IL-1RA | IVW | -0.12 (0.07) | 0.102 |
|  | Weighted Median | -0.14 (0.09) | 0.117 |
|  | MR Egger | -0.49 (0.33) | 0.149 |
| sIL-2Rα | IVW | -0.09 (0.08) | 0.269 |
|  | Weighted Median | -0.09 (0.10) | 0.266 |
|  | MR Egger | 0.24 (0.38) | 0.531 |
| IL-4 | IVW | 0.01 (0.04) | 0.810 |
|  | Weighted Median | 0.01 (0.06) | 0.800 |
|  | MR Egger | 0.02 (0.21) | 0.913 |
| IL-5 | IVW | -0.10 (0.06) | 0.130 |
|  | Weighted Median | -0.08 (0.09) | 0.367 |
|  | MR Egger | -0.43 (0.33) | 0.193 |
| IL-6 | IVW | 0.04 (0.04) | 0.285 |
|  | Weighted Median | 0.07 (0.06) | 0.260 |
|  | MR Egger | 0.04 (0.21) | 0.836 |
| IL-7 | IVW | -0.05 (0.07) | 0.423 |
|  | Weighted Median | 0.02 (0.09) | 0.834 |
|  | MR Egger | -0.23 (0.32) | 0.485 |
| IL-8 | IVW | -0.11 (0.06) | 0.090 |
|  | Weighted Median | -0.07 (0.09) | 0.408 |
|  | MR Egger | -0.74 (0.32) | 0.024 |
| IL-9 | IVW | -0.06 (0.06) | 0.311 |
|  | Weighted Median | -0.14 (0.09) | 0.125 |
|  | MR Egger | -0.01 (0.32) | 0.965 |
| IL-10 | IVW | 0.00 (0.04) | 0.907 |
|  | Weighted Median | -0.06 (0.06) | 0.317 |
|  | MR Egger | 0.26 (0.22) | 0.232 |
| IL-12 | IVW | 0.00 (0.04) | 0.983 |
|  | Weighted Median | -0.01 (0.06) | 0.902 |
|  | MR Egger | 0.33 (0.22) | 0.136 |
| IL-13 | IVW | -0.12 (0.06) | 0.063 |
|  | Weighted Median | -0.12 (0.09) | 0.332 |
|  | MR Egger | 0.05 (0.32) | 0.866 |
| IL-16 | IVW | 0.00 (0.06) | 0.974 |
|  | Weighted Median | -0.03 (0.09) | 0.765 |
|  | MR Egger | -0.30 (0.32) | 0.352 |
| IL-17 | IVW | -0.11 (0.06) | 0.070 |
|  | Weighted Median | -0.12 (0.09) | 0.349 |
|  | MR Egger | 0.05 (0.32) | 0.866 |
| IL-18 | IVW | -0.04 (0.13) | 0.556 |
|  | Weighted Median | -0.14 (0.09) | 0.115 |
|  | MR Egger | 0.02 (0.31) | 0.957 |
| CRP | IVW | -0.03 (0.02) | 0.100 |
|  | Weighted Median | -0.03 (0.02) | 0.052 |
|  | MR Egger | -0.19 (0.09) | 0.038* |
| MCP-1 | IVW | -0.07 (0.04) | 0.098 |
|  | Weighted Median | -0.07 (0.06) | 0.247 |
|  | MR Egger | -0.01 (0.23) | 0.975 |
| TNFa | IVW | -0.04 (0.06) | 0.504 |
|  | Weighted Median | -0.08 (0.09) | 0.402 |
|  | MR Egger | -0.32 (0.32) | 0.322 |
| Neutrophils | IVW | 0.00 (0.02) | 0.700 |
|  | Weighted Median | 0.02 (0,01) | 0.258 |
|  | MR Egger | -0.10 (0.09) | 0.262 |
| Lymphocytes | IVW | -0.04 (0.02) | 0.136 |
|  | Weighted Median | 0.01 (0.01) | 0.800 |
|  | MR Egger | -0.29 (0.11) | 0.010* |

**p*<0.05; ^†^*p*<0.003 (Bonferroni corrected for 19 exposures)

**Supplementary Table 13: ORs (95% CIs) for Bidirectional MR Associations of Major Depression with Immune-Regulatory Proteins**

| **Outcome** | **Method** | **B (SE)** | **P-value** |
| --- | --- | --- | --- |
| IL-1RA | IVW | -0.10 (0.30) | 0.747 |
|  | Weighted Median | -0.01 (0.43) | 0.975 |
|  | MR Egger | 2.97 (2.00) | 0.146 |
| sIL-2Rα | IVW | -0.04 (0.30) | 0.882 |
|  | Weighted Median | 0.27 (0.43) | 0.524 |
|  | MR Egger | 3.44 (1.99) | 0.109 |
| IL-4 | IVW | -0.03 (0.20) | 0.886 |
|  | Weighted Median | 0.04 (0.27) | 0.892 |
|  | MR Egger | -0.78 (1.35) | 0.579 |
| IL-5 | IVW | -0.15 (0.31) | 0.626 |
|  | Weighted Median | -0.11 (0.46) | 0.803 |
|  | MR Egger | 3.23 (2.08) | 0.129 |
| IL-6 | IVW | -0.16 (0.20) | 0.427 |
|  | Weighted Median | -0.28 (0.29) | 0.334 |
|  | MR Egger | 0.78 (1.34) | 0.566 |
| IL-7 | IVW | -0.01 (0.31) | 0.973 |
|  | Weighted Median | -0.11 (0.44) | 0.800 |
|  | MR Egger | 2.70 (2.07) | 0.201 |
| IL-8 | IVW | -0.43 (0.33) | 0.193 |
|  | Weighted Median | -0.45 (0.45) | 0.324 |
|  | MR Egger | -0.05 (2.23) | 0.983 |
| IL-9 | IVW | -0.08 (0.31) | 0.795 |
|  | Weighted Median | 0.10 (0.43) | 0.814 |
|  | MR Egger | 0.89 (2.06) | 0.667 |
| IL-10 | IVW | -0.35 (0.22) | 0.104 |
|  | Weighted Median | -0.43 (0.30) | 0.149 |
|  | MR Egger | 1.14 (1.43) | 0.403 |
| IL-12 | IVW | 0.04 (0.05) | 0.201 |
|  | Weighted Median | 0.08 (0.10) | 0.154 |
|  | MR Egger | 0.22 (0.19) | 0.443 |
| IL-13 | IVW | -0.26 (0.31) | 0.397 |
|  | Weighted Median | -0.45 (0.43) | 0.300 |
|  | MR Egger | 2.25 (2.02) | 0.274 |
| IL-16 | IVW | -0.32 (0.31) | 0.290 |
|  | Weighted Median | -0.44 (0.42) | 0.296 |
|  | MR Egger | -1.36 (2.05) | 0.511 |
| IL-17 | IVW | -0.25 (0.31) | 0.397 |
|  | Weighted Median | -0.45 (0.43) | 0.301 |
|  | MR Egger | 2.25 (2.02) | 0.273 |
| IL-18 | IVW | -0.36 (0.44) | 0.274 |
|  | Weighted Median | -0.34 (0.45) | 0.456 |
|  | MR Egger | 0.97 (2.19) | 0.659 |
| CRP | IVW | 0.09 (0.09) | 0.121 |
|  | Weighted Median | 0.12 (0.09) | 0.186 |
|  | MR Egger | 0.76 (0.58 | 0.167 |
| MCP-1 | IVW | -0.05 (0.21) | 0.822 |
|  | Weighted Median | 0.00 (0.30) | 0.996 |
|  | MR Egger | 2.39 (1.33) | 0.143 |
| TNFa | IVW | -0.08 (0.31) | 0.787 |
|  | Weighted Median | -0.01 (0.34) | 0.983 |
|  | MR Egger | 3.11 (2.06) | 0.140 |
| Neutrophils | IVW | -0.12 (0.20) | 0.378 |
|  | Weighted Median | -0.07 (0.10) | 0.447 |
|  | MR Egger | 0.11 (1.20) | 0.978 |
| Lymphocytes | IVW | 0.09 (0.10) | 0.319 |
|  | Weighted Median | 0.22 (0.17) | 0.684 |
|  | MR Egger | 1.23 (2.10) | 0.911 |

**p*<0.05; ^†^*p*<0.003 (Bonferroni corrected for 19 exposures)

**Supplementary Table 14: Results For Cochran’s Q Tests for Heterogeneity and MR Egger Intercept Tests for Horizontal Pleiotropy for the Association between Immune-Regulatory Proteins (*Trans* Variants) and Schizophrenia, Bipolar Disorder and Major Depression**

| **Outcome / Exposure** | **IVW** | | **MR Egger** | | | |
| --- | --- | --- | --- | --- | --- | --- |
|  | **Cochran’s Q (df)** | ***p-*value** | **Cochran’s Q (df)** | ***p-*value** | **MR Egger Intercept (SE)** | **Intercept *p-*value** |
| **Schizophrenia** | | | | | | |
| IL-1RA | 32.15 (12) | 0.001 | 31.33 (11) | 0.009 | 0.01 (0.02) | 0.601 |
| sIL-2Rα | 25.37 (13) | 0.020 | 24.94 (12) | 0.015 | 0.01 (0.01) | 0.657 |
| IL-4 | 20.55 (11) | 0.038 | 20.33 (10) | 0.026 | -0.01 (0.01) | 0.751 |
| IL-5 | 19.29 (10) | 0.037 | 18.01 (9) | 0.035 | -0.01 (0.02) | 0.444 |
| IL-7 | 14.50 (13) | 0.340 | 14.49 (12) | 0.271 | -0.00 (0.01) | 0.912 |
| IL-8 | 15.31 (13) | 0.288 | 15.29 (12) | 0.226 | -0.00 (0.01) | 0.891 |
| IL-9 | 10.71 (12) | 0.554 | 9.99 (11) | 0.531 | 0.01 (0.01) | 0.417 |
| IL-10 | 13.33 (14) | 0.501 | 12.11 (13) | 0.518 | -0.01 (0.01) | 0.290 |
| IL-12 | 12.19 (15) | 0.664 | 12.16 (14) | 0.593 | -0.01 (0.01) | 0.874 |
| IL-13 | 28.12 (17) | 0.043 | 27.52 (16) | 0.036 | -0.01 (0.01) | 0.562 |
| IL-16 | 8.41 (10) | 0.589 | 8.53 (11) | 0.665 | 0.00 (0.00) | 0.732 |
| IL-17 | 10.06 (12) | 0.610 | 9.81 (11) | 0.548 | 0.01 (0.01) | 0.622 |
| IL-18 | 11.71 (12) | 0.469 | 13.05 (13) | 0.444 | 0.01 (0.01) | 0.270 |
| CRP | 139.74 (44) | <0.001 | 139.74 (45) | <0.001 | 0.00 (0.01) | 0.993 |
| TNFa | 9.16 (9) | 0.422 | 8.82 (8) | 0.358 | 0.01 (0.01) | 0.590 |
| MCP-1 | 24.53 (23) | 0.375 | 24.21 (22) | 0.336 | 0.00 (0.01) | 0.594 |
| BDNF | 137.12 (58) | <0.001 | 137.06 (57) | <0.001 | 0.00 (0.01) | 0.875 |
| Neutrophils | 84.31 (50) | 0.002 | 84.10 (49) | 0.001 | 0.00 (0.00) | 0.726 |
| Lymphocytes | 108.93 (53) | <0.001 | 108.90 (52) | <0.001 | -0.00 (0.00) | 0.908 |
| **Bipolar Disorder** | | | | | | |
| IL-1RA | 70.88 (10) | <0.001 | 49.02 (9) | <0.001 | -0.05 (0.03) | 0.076 |
| sIL-2Rα | 26.53 (14) | 0.022 | 26.48 (13) | 0.015 | 0.00 (0.01) | 0.881 |
| IL-4 | 10.83 (12) | 0.544 | 9.88 (11) | 0.541 | 0.01 (0.01) | 0.352 |
| IL-5 | 14.02 (10) | 0.172 | 13.29 (9) | 0.150 | 0.01 (0.01) | 0.501 |
| IL-7 | 9.77 (12) | 0.635 | 9.49 (11) | 0.576 | 0.00 (0.01) | 0.611 |
| IL-8 | 21.31 (13) | 0.067 | 21.13 (12) | 0.048 | 0.00 (0.01) | 0.753 |
| IL-9 | 25.64 (12) | 0.012 | 25.52 (11) | 0.008 | 0.00 (0.02) | 0.826 |
| IL-10 | 24.33 (15) | 0.060 | 24.33 (14) | 0.042 | 0.00 (0.01) | 0.973 |
| IL-12 | 30.48 (15) | 0.010 | 30.13 (14) | 0.007 | 0.00 (0.01) | 0.693 |
| IL-13 | 32.67 (16) | 0.008 | 29.84 (15) | 0.001 | -0.01 (0.010 | 0.251 |
| IL-16 | 19.10 (10) | 0.039 | 16.26 (9) | 0.061 | -0.01 (0.01) | 0.241 |
| IL-17 | 10.35 (10) | 0.410 | 7.35 (9) | 0.600 | 0.02 (0.01) | 0.117 |
| IL-18 | 16.71 (18) | 0.543 | 16.68 (17) | 0.476 | 0.00 (0.01) | 0.859 |
| CRP | 226.16 (110) | <0.001 | 226.10 (109) | <0.001 | 0.00 (0.00) | 0.870 |
| TNFa | 8.53 (8) | 0.384 | 6.59 (7) | 0.472 | 0.01 (0.01) | 0.206 |
| MCP-1 | 26.90 (21) | 0.174 | 24.82 (20) | 0.208 | 0.01 (0.01) | 0.210 |
| BDNF | 72.28 (59) | 0.064 | 76.22 (58) | 0.054 | 0.00 (0.00) | 0.834 |
| Neutrophils | 106.45 (49) | <0.001 | 102.83 (48) | <0.001 | 0.01 (0.01) | 0.200 |
| Lymphocytes | 82.63 (54) | 0.007 | 79.73 (53) | 0.010 | -0.01 (0.01) | 0.170 |
| **Major Depression** | | | | | | |
| IL-1RA | 3.88 (11) | 0.973 | 3.88 (10) | 0.952 | -0.00 (0.01) | 0.988 |
| sIL-2Rα | 2.32 (15) | 0.999 | 1.92 (14) | 0.999 | -0.00 (0.00) | 0.533 |
| IL-4 | 1.58 (13) | 0.999 | 1.56 (12) | 0.999 | 0.00 (0.00) | 0.880 |
| IL-5 | 1.14 (11) | 0.999 | 1.12 (10) | 0.998 | 0.00 (0.01) | 0.892 |
| IL-7 | 3.83 (13) | 0.993 | 3.24 (12) | 0.994 | 0.01 (0.00) | 0.457 |
| IL-8 | 1.08 (13) | 0.999 | 1.05 (12) | 0.999 | 0.00 (0.01) | 0.857 |
| IL-9 | 1.49 (14) | 0.999 | 1.49 (13) | 0.999 | -0.00 (0.01) | 0.976 |
| IL-10 | 1.85 (15) | 0.999 | 1.84 (14) | 0.999 | -0.00 (0.00) | 0.979 |
| IL-12 | 1.82 (16) | 0.999 | 1.81 (15) | 0.999 | -0.00 (0.00) | 0.910 |
| IL-13 | 5.63 (17) | 0.995 | 5.32 (16) | 0.994 | -0.00 (0.00) | 0.583 |
| IL-16 | 1.57 (12) | 0.999 | 1.55 (11) | 0.996 | 0.00 (0.00) | 0.904 |
| IL-17 | 3.36 (15) | 0.999 | 3.36 (14) | 0.998 | -0.00 (0.00) | 0.976 |
| IL-18 | 2.60 (17) | 0.999 | 2.56 (16) | 0.999 | 0.00 (0.00) | 0.833 |
| CRP | 28.93 (119) | 0.999 | 28.91 (118) | 0.999 | -0.00 (0.00) | 0.901 |
| TNFa | 1.93 (10) | 0.997 | 1.68 (9) | 0.996 | 0.00 (0.00) | 0.628 |
| MCP-1 | 4.35 (26) | 0.999 | 4.26 (25) | 0.999 | -0.00 (0.00) | 0.766 |
| BDNF | 14.25 (60) | 0.999 | 14.24 (59) | 0.999 | 0.00 (0.00) | 0.956 |
| Neutrophils | 11.34 (53) | 0.999 | 11.32 (52) | 0.999 | -0.00 (0.00) | 0.883 |
| Lymphocytes | 13.77 (55) | 0.999 | 13.13 (54) | 0.999 | -0.00 (0.00) | 0.424 |

**Supplementary Table 15: Results For Cochran’s Q Tests for Heterogeneity and MR Egger Intercept Tests for Horizontal Pleiotropy for the Bidirectional MR Associations of Schizophrenia, Bipolar Disorder and Major Depression with Immune-Regulatory Proteins**

| **Exposure / Outcome** | **IVW** | | **MR Egger** | | | |
| --- | --- | --- | --- | --- | --- | --- |
|  | **Cochran’s Q (df)** | ***p-*value** | **Cochran’s Q (df)** | ***p-*value** | **MR Egger Intercept (SE)** | **Intercept *p-*value** |
| **Schizophrenia as Exposure** | | | | | | |
| IL-1RA | 167.94 (143) | 0.076 | 167.80 (142) | 0.069 | 0.00 (0.01) | 0.738 |
| sIL-2Rα | 163.06 (143) | 0.120 | 160.32 (142) | 0.140 | 0.02 (0.01) | 0.121 |
| IL-4 | 107.98 (143) | 0.054 | 107.99 (142) | 0.047 | -0.00 (0.01) | 0.994 |
| IL-5 | 87.34 (143) | 0.439 | 85.43 (142) | 0.467 | 0.01 (0.07) | 0.941 |
| IL-6 | 145.29 (143) | 0.431 | 145.25 (142) | 0.409 | 0.00 (0.01) | 0.840 |
| IL-7 | 107.89 (143) | 0.056 | 107.89 (142) | 0.048 | 0.05 (0.06) | 0.423 |
| IL-8 | 97.34 (143) | 0.189 | 96.81 (142) | 0.179 | -0.01 (0.02) | 0.493 |
| IL-9 | 90.39 (143) | 0.351 | 90.22 (142) | 0.329 | -0.01 (0.01) | 0.694 |
| IL-10 | 98.91 (143) | 0.161 | 98.56 (142) | 0.149 | 0.01 (0.04) | 0.711 |
| IL-12 | 160.27 (143) | 0.153 | 160.01 (142) | 0.143 | 0.00 (0.01) | 0.634 |
| IL-13 | 97.06 (143) | 0.195 | 97.06 (142) | 0.175 | 0.04 (0.06) | 0.494 |
| IL-16 | 94.64 (143) | 0.248 | 91.25 (142) | 0.302 | -0.03 (0.01) | 0.079 |
| IL-17 | 149.02 (143) | 0.348 | 148.86 (142) | 0.330 | 0.00 (0.01) | 0.700 |
| IL-18 | 68.97 (144) | 0.910 | 67.64 (142) | 0.917 | -0.07 (0.05) | 0.248 |
| CRP | 82.63 (133) | 0.999 | 82.63 (132) | 0.999 | 0.00 (0.01) | 0.989 |
| TNFa | 163.55 (143) | 0.115 | 161.96 (142) | 0.121 | -0.01 (0.01) | 0.239 |
| MCP-1 | 139.81 (143) | 0.560 | 138.39 (142) | 0.570 | 0.01 (0.01) | 0.236 |
| Neutrophils | 92.23 (143) | 0.332 | 89.96 (142) | 0.220 | 0.04 (0.01) | 0.031 |
| Lymphocytes | 89.12 (143) | 0.298 | 90.33 (142) | 0.301 | 0.03 (0.01) | 0.049 |
| **Bipolar Disorder as Exposure** | | | | | | |
| IL-1RA | 44.42 (40) | 0.291 | 42.96 (39) | 0.305 | 0.03 (0.02) | 0.257 |
| sIL-2Rα | 56.54 (40) | 0.043 | 55.48 (39) | 0.042 | -0.02 (0.03) | 0.392 |
| IL-4 | 30.64 (40) | 0.856 | 30.64 (39) | 0.828 | 0.00 (0.01) | 0.951 |
| IL-5 | 28.48 (40) | 0.913 | 27.38 (39) | 0.919 | 0.02 (0.02) | 0.301 |
| IL-6 | 30.92 (40) | 0.848 | 30.92 (39) | 0.819 | 0.00 (0.01) | 0.979 |
| IL-7 | 36.26 (40) | 0.639 | 35.95 (39) | 0.609 | 0.01 (0.02) | 0.580 |
| IL-8 | 37.53 (40) | 0.582 | 33.38 (39) | 0.724 | 0.04 (0.02) | 0.048 |
| IL-9 | 39.51 (40) | 0.493 | 39.48 (39) | 0.448 | 0.00 (0.02) | 0.876 |
| IL-10 | 26.11 (40) | 0.956 | 24.52 (39) | 0.966 | -0.02 (0.01) | 0.214 |
| IL-12 | 44.53 (40) | 0.287 | 41.95 (39) | 0.344 | -0.02 (0.01) | 0.129 |
| IL-13 | 37.39 (40) | 0.589 | 37.09 (39) | 0.557 | -0.01 (0.02) | 0.588 |
| IL-16 | 38.17 (40) | 0.553 | 27.26 (39) | 0.549 | 0.02 (0.02) | 0.347 |
| IL-17 | 37.39 (40) | 0.589 | 37.09 (39) | 0.557 | -0.01 (0.02) | 0.588 |
| IL-18 | 38.96 (40) | 0.517 | 38.70 (39) | 0.484 | -0.01 (0.02) | 0.609 |
| CRP | 79.94 (36) | <0.001 | 73.35 (35) | <0.001 | 0.01 (0.01) | 0.085 |
| TNFa | 36.68 (40) | 0.621 | 35.89 (39) | 0.612 | 0.02 (0.02) | 0.380 |
| MCP-1 | 46.25 (40) | 0.230 | 46.15 (39) | 0.201 | 0.00 (0.02) | 0.768 |
| Neutrophils | 159.14 (40) | <0.001 | 153.05 (39) | <0.001 | 0.01 (0.01) | 0.220 |
| Lymphocytes | 248.84 (40) | <0.001 | 215.85 (39) | <0.001 | 0.02 (0.01) | 0.019 |
| **Major Depression as Exposure** | | | | | | |
| IL-1RA | 36.52 (36) | 0.444 | 34.11 (35) | 0.511 | -0.04 (0.03) | 0.129 |
| sIL-2Rα | 37.18 (36) | 0.415 | 34.06 (35) | 0.513 | -0.05 (0.03) | 0.087 |
| IL-4 | 32.23 (36) | 0.601 | 32.93 (35) | 0.568 | 0.01 (0.02) | 0.589 |
| IL-5 | 35.41 (36) | 0.496 | 32.70 (35) | 0.579 | -0.05 (0.02) | 0.032 |
| IL-6 | 31.64 (36) | 0.676 | 31.14 (35) | 0.655 | -0.01 (0.02) | 0.484 |
| IL-7 | 33.57 (36) | 0.585 | 31.81 (35) | 0.623 | -0.03 (0.03) | 0.193 |
| IL-8 | 42.32 (36) | 0.212 | 42.28 (35) | 0.186 | -0.01 (0.03) | 0.862 |
| IL-9 | 37.20 (36) | 0.413 | 36.96 (35) | 0.378 | -0.01 (0.03) | 0.637 |
| IL-10 | 39.07 (36) | 0.333 | 37.87 (35) | 0.340 | -0.02 (0.02) | 0.299 |
| IL-12 | 42.11 (36) | 0.661 | 40.76 (35) | 0.687 | -0.05 (0.02) | 0.041 |
| IL-13 | 29.61 (36) | 0.765 | 28.05 (35) | 0.792 | -0.04 (0.03) | 0.219 |
| IL-16 | 26.20 (36) | 0.885 | 25.94 (35) | 0.867 | 0.01 (0.03) | 0.612 |
| IL-17 | 29.61 (36) | 0.765 | 28.05 (35) | 0.792 | -0.04 (0.03) | 0.219 |
| IL-18 | 41.82 (36) | 0.233 | 41.36 (35) | 0.213 | -0.02 (0.03) | 0.541 |
| CRP | 65.90 (33) | 0.009 | 63.43 (32) | 0.008 | -0.01 (0.01) | 0.272 |
| TNFa | 30.36 (36) | 0.733 | 27.90 (35) | 0.798 | -0.05 (0.03) | 0.127 |
| MCP-1 | 38.04 (36) | 0.377 | 34.66 (35) | 0.484 | -0.04 (0.02) | 0.075 |
| Neutrophils | 33.21 (36) | 0.446 | 35.12 (35) | 0.201 | -0.06 (0.01) | 0.012 |
| Lymphocytes | 39.12 (36) | 0.318 | 37.87 (35) | 0.398 | -0.05 (0.01) | 0.041 |

**Supplementary Table 16: Results of MR PRESSO Analysis for Associations between Immune-Regulatory Proteins and Schizophrenia, Bipolar Disorder and Major Depression**

| **Outcome / Risk Factor** | **MR-PRESSO Global Test** | | **Outlier-Corrected IVW** | | **Distortion Test** | |
| --- | --- | --- | --- | --- | --- | --- |
|  | **RSS** | ***p*-value** | **β (SE)** | ***p*-value** | **Coefficient** | ***p*-value** |
| **Schizophrenia** | | | | | | |
| IL-1RA | 37.98 | 0.001 | 0.01 (0.03) | 0.875 | -380.84 | 0.102 |
| sIL-2Rα | 37.81 | 0.004 | 0.02 (0.02) | 0.398 | 49.82 | 0.421 |
| IL-4 | 32.44 | 0.170 | * | * | * | * |
| IL-5 | 22.59 | 0.079 | * | * | * | * |
| IL-7 | 27.09 | 0.157 | * | * | * | * |
| IL-8 | 20.40 | 0.199 | * | * | * | * |
| IL-9 | 14.32 | 0.601 | * | * | * | * |
| IL-10 | 18.36 | 0.501 | * | * | * | * |
| IL-12 | 24.34 | 0.299 | * | * | * | * |
| IL-13 | 35.02 | 0.082 | * | * | * | * |
| IL-16 | 9.57 | 0.784 | * | * | * | * |
| IL-17 | 33.03 | 0.017 | -0.03 (0.03) | 0.305 | 86.88 | 0.664 |
| IL-18 | 20.15 | 0.408 | * | * | * | * |
| CRP | 292.48 | <0.001 | -0.11 (0.03) | <0.001 | 31.11 | 0.372 |
| BDNF | 156.03 | <0.001 | -0.01 (0.01) | 0.847 | 185.30 | 0.220 |
| TNFa | 11.16 | 0.436 | * | * | * | * |
| MCP-1 | 28.34 | 0.340 | * | * | * | * |
| Neutrophils | 156.95 | <0.001 | 0.05 (0.04) | 0.179 | 58.29 | 0.181 |
| Lymphocytes | 269.09 | <0.001 | -0.04 (0.04) | 0.280 | -45.66 | 0.346 |
| **Bipolar Disorder** | | | | | | |
| IL-1RA | 86.89 | <0.001 | 0.02 (0.03) | 0.391 | -131.49 | 0.295 |
| sIL-2Rα | 33.20 | 0.037 | 0.025 (0.02) | 0.132 | -44.26 | 0.572 |
| IL-4 | 14.93 | 0.666 | * | * | * | * |
| IL-5 | 15.83 | 0.285 | * | * | * | * |
| IL-7 | 13.91 | 0.686 | * | * | * | * |
| IL-8 | 26.11 | 0.776 | * | * | * | * |
| IL-9 | 41.57 | 0.001 | -0.01 (0.03) | 0.763 | 166.76 | 0.175 |
| IL-10 | 28.87 | 0.088 | * | * | * | * |
| IL-12 | 35.19 | 0.052 | * | * | * | * |
| IL-13 | 37.83 | 0.021 | * | * | * | * |
| IL-16 | 27.16 | 0.080 | * | * | * | * |
| IL-17 | 20.68 | 0.218 | * | * | * | * |
| IL-18 | 18.40 | 0.563 | * | * | * | * |
| CRP | 305.74 | <0.001 | -0.04 (0.03) | 0.207 | 60.85 | 0.593 |
| BDNF | 94.74 | 0.087 | * | * | * | * |
| TNFa | 10.89 | 0.405 | * | * | * | * |
| MCP-1 | 30.22 | 0.171 | * | * | * | * |
| Neutrophils | 219.23 | <0.001 | 0.02 (0.04) | 0.705 | 9.27 | 0.974 |
| Lymphocytes | 150.53 | <0.001 | -0.05 (0.04) | 0.161 | -15.42 | 0.798 |
| **Major Depression** | | | | | | |
| IL-1RA | 4.79 | 0.991 | * | * | * | * |
| sIL-2Rα | 3.90 | 0.999 | * | * | * | * |
| IL-4 | 4.25 | 0.999 | * | * | * | * |
| IL-5 | 2.39 | 0.999 | * | * | * | * |
| IL-7 | 5.25 | 0.999 | * | * | * | * |
| IL-8 | 1.51 | 0.999 | * | * | * | * |
| IL-9 | 2.28 | 0.999 | * | * | * | * |
| IL-10 | 2.57 | 0.999 | * | * | * | * |
| IL-12 | 2.05 | 0.999 | * | * | * | * |
| IL-13 | 6.96 | 0.999 | * | * | * | * |
| IL-16 | 1.72 | 0.999 | * | * | * | * |
| IL-17 | 4.23 | 0.999 | * | * | * | * |
| IL-18 | 4.93 | 0.999 | * | * | * | * |
| CRP | 33.69 | 0.997 | * | * | * | * |
| BDNF | 17.99 | 0.998 | * | * | * | * |
| TNFa | 2.35 | 0.996 | * | * | * | * |
| MCP-1 | 28.34 | 0.342 | * | * | * | * |
| Neutrophils | 24.42 | 0.999 | * | * | * | * |
| Lymphocytes | 21.41 | 0.999 | * | * | * | * |

*****test not conducted due to no evidence of horizontal pleiotropy

**Supplementary Table 17: Results of MR PRESSO Analysis for Bidirectional Analyses of Schizophrenia, Bipolar Disorder and Major Depression with Immune-Regulatory Proteins**

| **Outcome** | **MR-PRESSO Global Test** | | **Outlier-Corrected IVW** | | **Distortion Test** | |
| --- | --- | --- | --- | --- | --- | --- |
|  | **RSS** | ***p*-value** | **β (SE)** | ***p*-value** | **Coefficient** | ***p*-value** |
| **Schizophrenia as Exposure** | | | | | | |
| IL-1RA | 108.77 | 0.386 | * | * | * | * |
| sIL-2Rα | 110.27 | 0.329 | * | * | * | * |
| IL-4 | 126.38 | 0.090 | * | * | * | * |
| IL-5 | 104.61 | 0.456 | * | * | * | * |
| IL-6 | 112.14 | 0.266 | * | * | * | * |
| IL-7 | 122.14 | 0.112 | * | * | * | * |
| IL-8 | 117.97 | 0.179 | * | * | * | * |
| IL-9 | 112.21 | 0.261 | * | * | * | * |
| IL-10 | 112.72 | 0.259 | * | * | * | * |
| IL-12 | 139.98 | <0.001 | -0.08 (0.04) | 0.105 | 49.98 | 0.546 |
| IL-13 | 116.05 | 0.208 | * | * | * | * |
| IL-16 | 110.59 | 0.349 | * | * | * | * |
| IL-17 | 116.05 | 0.219 | * | * | * | * |
| IL-18 | 82.53 | 0.937 | * | * | * | * |
| CRP | 292.19 | <0.001 | 0.02 (0.02) | 0.436 | -91.16 | 0.726 |
| TNFa | 124.15 | 0.081 | * | * | * | * |
| MCP-1 | 101.02 | 0.580 | * | * | * | * |
| **Bipolar Disorder as Exposure** | | | | | | |
| IL-1RA | 48.31 | 0.543 | * | * | * | * |
| sIL-2Rα | 65.90 | 0.065 | * | * | * | * |
| IL-4 | 35.01 | 0.950 | * | * | * | * |
| IL-5 | 39.74 | 0.838 | * | * | * | * |
| IL-6 | 33.37 | 0.966 | * | * | * | * |
| IL-7 | 41.74 | 0.791 | * | * | * | * |
| IL-8 | 49.40 | 0.507 | * | * | * | * |
| IL-9 | 45.73 | 0.638 | * | * | * | * |
| IL-10 | 29.56 | 0.988 | * | * | * | * |
| IL-12 | 54.60 | 0.297 | * | * | * | * |
| IL-13 | 46.30 | 0.612 | * | * | * | * |
| IL-16 | 44.03 | 0.710 | * | * | * | * |
| IL-17 | 46.29 | 0.621 | * | * | * | * |
| IL-18 | 52.57 | 0.384 | * | * | * | * |
| CRP | 106.51 | <0.001 | -0.03 (0.01) | 0.024* | 42.52 | 0.504 |
| TNFa | 46.85 | 0.601 | * | * | * | * |
| MCP-1 | 58.04 | 0.207 | * | * | * | * |
| Neutrophils | 58.04 | 0.207 | * | * | * | * |
| Lymphocytes | 279.07 | <0.001 | -0.01 (0.01) | 0.458 | 55.845 | 0.335 |
| **Major Depression as Exposure** | | | | | | |
| IL-1RA | 39.24 | 0.566 | * | * | * | * |
| sIL-2Rα | 40.37 | 0.511 | * | * | * | * |
| IL-4 | 38.55 | 0.587 | * | * | * | * |
| IL-5 | 38.51 | 0.586 | * | * | * | * |
| IL-6 | 34.39 | 0.735 | * | * | * | * |
| IL-7 | 36.43 | 0.681 | * | * | * | * |
| IL-8 | 44.84 | 0.302 | * | * | * | * |
| IL-9 | 41.82 | 0.452 | * | * | * | * |
| IL-10 | 42.08 | 0.461 | * | * | * | * |
| IL-12 | 64.21 | 0.021 | 0.06 (0.08) | 0.234 | 0.231 | 0.987 |
| IL-13 | 33.19 | 0.798 | * | * | * | * |
| IL-16 | 29.99 | 0.896 | * | * | * | * |
| IL-17 | 33.19 | 0.789 | * | * | * | * |
| IL-18 | 44.87 | 0.344 | * | * | * | * |
| CRP | 71.62 | 0.002 | 0.17 (0.14) | 0.201 | -0.872 | 0.987 |
| TNFa | 36.53 | 0.651 | * | * | * | * |
| MCP-1 | 46.59 | 0.260 | * | * | * | * |

*****test not conducted due to no evidence of horizontal pleiotropy

**Supplementary Table 18: I_2GX_ Statistics to Examine for Potential Violation of NOME Assumption for MR Egger Analyses**

| **Exposure** | **I^2^_GX_ of SNP-Exposure Associations** |
| --- | --- |
| IL-1RA | 0.647 |
| sIL-2Rα | 0.900 |
| IL-4 | 0.791 |
| IL-5 | 0.776 |
| IL-6 | 0.813 |
| IL-7 | 0.867 |
| IL-8 | 0.770 |
| IL-9 | 0.758 |
| IL-10 | 0.877 |
| IL-12 | 0.989 |
| IL-13 | 0.894 |
| IL-16 | 0.909 |
| IL-17 | 0.760 |
| IL-18 | 0.845 |
| CRP | 0.953 |
| TNFa | 0.822 |
| MCP-1 | 0.846 |
| BDNF | 0.943 |
| Neutrophils | 0.948 |
| Lymphocytes | 0.934 |
| Schizophrenia (as exposure) | 0.000 |
| Bipolar Disorder (as exposure) | 0.360 |
| Depression (as exposure) | 0.120 |

**Reference**

Ahola-Olli, A.V., Wurtz, P., Havulinna, A.S., Aalto, K., Pitkanen, N., Lehtimaki, T., Kahonen, M., Lyytikainen, L.P., Raitoharju, E., Seppala, I., Sarin, A.P., Ripatti, S., Palotie, A., Perola, M., Viikari, J.S., Jalkanen, S., Maksimow, M., Salomaa, V., Salmi, M., Kettunen, J., Raitakari, O.T., 2017. Genome-wide Association Study Identifies 27 Loci Influencing Concentrations of Circulating Cytokines and Growth Factors. Am J Hum Genet 100, 40-50.

Astle, W.J., Elding, H., Jiang, T., Allen, D., Ruklisa, D., Mann, A.L., Mead, D., Bouman, H., Riveros-Mckay, F., Kostadima, M.A., Lambourne, J.J., Sivapalaratnam, S., Downes, K., Kundu, K., Bomba, L., Berentsen, K., Bradley, J.R., Daugherty, L.C., Delaneau, O., Freson, K., Garner, S.F., Grassi, L., Guerrero, J., Haimel, M., Janssen-Megens, E.M., Kaan, A., Kamat, M., Kim, B., Mandoli, A., Marchini, J., Martens, J.H.A., Meacham, S., Megy, K., O'Connell, J., Petersen, R., Sharifi, N., Sheard, S.M., Staley, J.R., Tuna, S., van der Ent, M., Walter, K., Wang, S.Y., Wheeler, E., Wilder, S.P., Iotchkova, V., Moore, C., Sambrook, J., Stunnenberg, H.G., Di Angelantonio, E., Kaptoge, S., Kuijpers, T.W., Carrillo-de-Santa-Pau, E., Juan, D., Rico, D., Valencia, A., Chen, L., Ge, B., Vasquez, L., Kwan, T., Garrido-Martin, D., Watt, S., Yang, Y., Guigo, R., Beck, S., Paul, D.S., Pastinen, T., Bujold, D., Bourque, G., Frontini, M., Danesh, J., Roberts, D.J., Ouwehand, W.H., Butterworth, A.S., Soranzo, N., 2016. The Allelic Landscape of Human Blood Cell Trait Variation and Links to Common Complex Disease. Cell 167, 1415-1429 e1419.

Bowden, J., Davey Smith, G., Burgess, S., 2015. Mendelian randomization with invalid instruments: effect estimation and bias detection through Egger regression. Int J Epidemiol 44, 512-525.

Bowden, J., Davey Smith, G., Haycock, P.C., Burgess, S., 2016. Consistent Estimation in Mendelian Randomization with Some Invalid Instruments Using a Weighted Median Estimator. Genet Epidemiol 40, 304-314.

Bowden, J., Del Greco, M.F., Minelli, C., Davey Smith, G., Sheehan, N., Thompson, J., 2017. A framework for the investigation of pleiotropy in two-sample summary data Mendelian randomization. Stat Med 36, 1783-1802.

Burgess, S., 2014. Sample size and power calculations in Mendelian randomization with a single instrumental variable and a binary outcome. Int J Epidemiol 43, 922-929.

Burgess, S., Butterworth, A., Thompson, S.G., 2013. Mendelian randomization analysis with multiple genetic variants using summarized data. Genet Epidemiol 37, 658-665.

Collaboration, I.R.G.C.E.R.F., Sarwar, N., Butterworth, A.S., Freitag, D.F., Gregson, J., Willeit, P., Gorman, D.N., Gao, P., Saleheen, D., Rendon, A., Nelson, C.P., Braund, P.S., Hall, A.S., Chasman, D.I., Tybjaerg-Hansen, A., Chambers, J.C., Benjamin, E.J., Franks, P.W., Clarke, R., Wilde, A.A., Trip, M.D., Steri, M., Witteman, J.C., Qi, L., van der Schoot, C.E., de Faire, U., Erdmann, J., Stringham, H.M., Koenig, W., Rader, D.J., Melzer, D., Reich, D., Psaty, B.M., Kleber, M.E., Panagiotakos, D.B., Willeit, J., Wennberg, P., Woodward, M., Adamovic, S., Rimm, E.B., Meade, T.W., Gillum, R.F., Shaffer, J.A., Hofman, A., Onat, A., Sundstrom, J., Wassertheil-Smoller, S., Mellstrom, D., Gallacher, J., Cushman, M., Tracy, R.P., Kauhanen, J., Karlsson, M., Salonen, J.T., Wilhelmsen, L., Amouyel, P., Cantin, B., Best, L.G., Ben-Shlomo, Y., Manson, J.E., Davey-Smith, G., de Bakker, P.I., O'Donnell, C.J., Wilson, J.F., Wilson, A.G., Assimes, T.L., Jansson, J.O., Ohlsson, C., Tivesten, A., Ljunggren, O., Reilly, M.P., Hamsten, A., Ingelsson, E., Cambien, F., Hung, J., Thomas, G.N., Boehnke, M., Schunkert, H., Asselbergs, F.W., Kastelein, J.J., Gudnason, V., Salomaa, V., Harris, T.B., Kooner, J.S., Allin, K.H., Nordestgaard, B.G., Hopewell, J.C., Goodall, A.H., Ridker, P.M., Holm, H., Watkins, H., Ouwehand, W.H., Samani, N.J., Kaptoge, S., Di Angelantonio, E., Harari, O., Danesh, J., 2012. Interleukin-6 receptor pathways in coronary heart disease: a collaborative meta-analysis of 82 studies. Lancet 379, 1205-1213.

Ferreira, R.C., Freitag, D.F., Cutler, A.J., Howson, J.M., Rainbow, D.B., Smyth, D.J., Kaptoge, S., Clarke, P., Boreham, C., Coulson, R.M., Pekalski, M.L., Chen, W.M., Onengut-Gumuscu, S., Rich, S.S., Butterworth, A.S., Malarstig, A., Danesh, J., Todd, J.A., 2013. Functional IL6R 358Ala allele impairs classical IL-6 receptor signaling and influences risk of diverse inflammatory diseases. PLoS genetics 9, e1003444.

Georgakis, M.K., Malik, R., Gill, D., Franceschini, N., Sudlow, C.L.M., Dichgans, M., Invent Consortium, C.I.W.G., 2020. Interleukin-6 Signaling Effects on Ischemic Stroke and Other Cardiovascular Outcomes: A Mendelian Randomization Study. Circulation. Genomic and precision medicine 13, e002872.

Ligthart, S., Vaez, A., Võsa, U., Stathopoulou, M.G., de Vries, P.S., Prins, B.P., Van der Most, P.J., Tanaka, T., Naderi, E., Rose, L.M., Wu, Y., Karlsson, R., Barbalic, M., Lin, H., Pool, R., Zhu, G., Macé, A., Sidore, C., Trompet, S., Mangino, M., Sabater-Lleal, M., Kemp, J.P., Abbasi, A., Kacprowski, T., Verweij, N., Smith, A.V., Huang, T., Marzi, C., Feitosa, M.F., Lohman, K.K., Kleber, M.E., Milaneschi, Y., Mueller, C., Huq, M., Vlachopoulou, E., Lyytikäinen, L.P., Oldmeadow, C., Deelen, J., Perola, M., Zhao, J.H., Feenstra, B., Amini, M., Lahti, J., Schraut, K.E., Fornage, M., Suktitipat, B., Chen, W.M., Li, X., Nutile, T., Malerba, G., Luan, J., Bak, T., Schork, N., Del Greco M, F., Thiering, E., Mahajan, A., Marioni, R.E., Mihailov, E., Eriksson, J., Ozel, A.B., Zhang, W., Nethander, M., Cheng, Y.C., Aslibekyan, S., Ang, W., Gandin, I., Yengo, L., Portas, L., Kooperberg, C., Hofer, E., Rajan, K.B., Schurmann, C., den Hollander, W., Ahluwalia, T.S., Zhao, J., Draisma, H.H.M., Ford, I., Timpson, N., Teumer, A., Huang, H., Wahl, S., Liu, Y., Huang, J., Uh, H.W., Geller, F., Joshi, P.K., Yanek, L.R., Trabetti, E., Lehne, B., Vozzi, D., Verbanck, M., Biino, G., Saba, Y., Meulenbelt, I., O'Connell, J.R., Laakso, M., Giulianini, F., Magnusson, P.K.E., Ballantyne, C.M., Hottenga, J.J., Montgomery, G.W., Rivadineira, F., Rueedi, R., Steri, M., Herzig, K.H., Stott, D.J., Menni, C., Frånberg, M., St Pourcain, B., Felix, S.B., Pers, T.H., Bakker, S.J.L., Kraft, P., Peters, A., Vaidya, D., Delgado, G., Smit, J.H., Großmann, V., Sinisalo, J., Seppälä, I., Williams, S.R., Holliday, E.G., Moed, M., Langenberg, C., Räikkönen, K., Ding, J., Campbell, H., Sale, M.M., Chen, Y.I., James, A.L., Ruggiero, D., Soranzo, N., Hartman, C.A., Smith, E.N., Berenson, G.S., Fuchsberger, C., Hernandez, D., Tiesler, C.M.T., Giedraitis, V., Liewald, D., Fischer, K., Mellström, D., Larsson, A., Wang, Y., Scott, W.R., Lorentzon, M., Beilby, J., Ryan, K.A., Pennell, C.E., Vuckovic, D., Balkau, B., Concas, M.P., Schmidt, R., Mendes de Leon, C.F., Bottinger, E.P., Kloppenburg, M., Paternoster, L., Boehnke, M., Musk, A.W., Willemsen, G., Evans, D.M., Madden, P.A.F., Kähönen, M., Kutalik, Z., Zoledziewska, M., Karhunen, V., Kritchevsky, S.B., Sattar, N., Lachance, G., Clarke, R., Harris, T.B., Raitakari, O.T., Attia, J.R., van Heemst, D., Kajantie, E., Sorice, R., Gambaro, G., Scott, R.A., Hicks, A.A., Ferrucci, L., Standl, M., Lindgren, C.M., Starr, J.M., Karlsson, M., Lind, L., Li, J.Z., Chambers, J.C., Mori, T.A., de Geus, E.J.C.N., Heath, A.C., Martin, N.G., Auvinen, J., Buckley, B.M., de Craen, A.J.M., Waldenberger, M., Strauch, K., Meitinger, T., Scott, R.J., McEvoy, M., Beekman, M., Bombieri, C., Ridker, P.M., Mohlke, K.L., Pedersen, N.L., Morrison, A.C., Boomsma, D.I., Whitfield, J.B., Strachan, D.P., Hofman, A., Vollenweider, P., Cucca, F., Jarvelin, M.R., Jukema, J.W., Spector, T.D., Hamsten, A., Zeller, T., Uitterlinden, A.G., Nauck, M., Gudnason, V., Qi, L., Grallert, H., Borecki, I.B., Rotter, J.I., März, W., Wild, P.S., Lokki, M.L., Boyle, M., Salomaa, V., Melbye, M., Eriksson, J.G., Wilson, J.F., Penninx, B.W.J.H., Becker, D.M., Worrall, B.B., Gibson, G., Krauss, R.M., Ciullo, M., Zaza, G., Wareham, N.J., Oldehinkel, A.J., Palmer, L.J., Murray, S.S., Pramstaller, P.P., Bandinelli, S., Heinrich, J., Ingelsson, E., Deary, I.J., Mägi, R., Vandenput, L., van der Harst, P., Desch, K.C., Kooner, J.S., Ohlsson, C., Hayward, C., Lehtimäki, T., Shuldiner, A.R., Arnett, D.K., Beilin, L.J., Robino, A., Froguel, P., Pirastu, M., Jess, T., Koenig, W., Loos, R.J.F., Evans, D.A., Schmidt, H., Smith, G.D., Slagboom, P.E., Eiriksdottir, G., Morris, A.P., Psaty, B.M., Tracy, R.P., Nolte, I.M., Boerwinkle, E., Visvikis-Siest, S., Reiner, A.P., Gross, M., Bis, J.C., Franke, L., Franco, O.H., Benjamin, E.J., Chasman, D.I., Dupuis, J., Snieder, H., Dehghan, A., Alizadeh, B.Z., Study, L.C., Group, C.I.W., 2018. Genome Analyses of >200,000 Individuals Identify 58 Loci for Chronic Inflammation and Highlight Pathways that Link Inflammation and Complex Disorders. Am J Hum Genet 103, 691-706.

Mullins, N., Forstner, A.J., O'Connell, K.S., Coombes, B., Coleman, J.R.I., Qiao, Z., Als, T.D., Bigdeli, T.B., Borte, S., Bryois, J., Charney, A.W., Drange, O.K., Gandal, M.J., Hagenaars, S.P., Ikeda, M., Kamitaki, N., Kim, M., Krebs, K., Panagiotaropoulou, G., Schilder, B.M., Sloofman, L.G., Steinberg, S., Trubetskoy, V., Winsvold, B.S., Won, H.H., Abramova, L., Adorjan, K., Agerbo, E., Al Eissa, M., Albani, D., Alliey-Rodriguez, N., Anjorin, A., Antilla, V., Antoniou, A., Awasthi, S., Baek, J.H., Baekvad-Hansen, M., Bass, N., Bauer, M., Beins, E.C., Bergen, S.E., Birner, A., Bocker Pedersen, C., Boen, E., Boks, M.P., Bosch, R., Brum, M., Brumpton, B.M., Brunkhorst-Kanaan, N., Budde, M., Bybjerg-Grauholm, J., Byerley, W., Cairns, M., Casas, M., Cervantes, P., Clarke, T.K., Cruceanu, C., Cuellar-Barboza, A., Cunningham, J., Curtis, D., Czerski, P.M., Dale, A.M., Dalkner, N., David, F.S., Degenhardt, F., Djurovic, S., Dobbyn, A.L., Douzenis, A., Elvsashagen, T., Escott-Price, V., Ferrier, I.N., Fiorentino, A., Foroud, T.M., Forty, L., Frank, J., Frei, O., Freimer, N.B., Frisen, L., Gade, K., Garnham, J., Gelernter, J., Giortz Pedersen, M., Gizer, I.R., Gordon, S.D., Gordon-Smith, K., Greenwood, T.A., Grove, J., Guzman-Parra, J., Ha, K., Haraldsson, M., Hautzinger, M., Heilbronner, U., Hellgren, D., Herms, S., Hoffmann, P., Holmans, P.A., Huckins, L., Jamain, S., Johnson, J.S., Kalman, J.L., Kamatani, Y., Kennedy, J.L., Kittel-Schneider, S., Knowles, J.A., Kogevinas, M., Koromina, M., Kranz, T.M., Kranzler, H.R., Kubo, M., Kupka, R., Kushner, S.A., Lavebratt, C., Lawrence, J., Leber, M., Lee, H.J., Lee, P.H., Levy, S.E., Lewis, C., Liao, C., Lucae, S., Lundberg, M., MacIntyre, D.J., Magnusson, S.H., Maier, W., Maihofer, A., Malaspina, D., Maratou, E., Martinsson, L., Mattheisen, M., McCarroll, S.A., McGregor, N.W., McGuffin, P., McKay, J.D., Medeiros, H., Medland, S.E., Millischer, V., Montgomery, G.W., Moran, J.L., Morris, D.W., Muhleisen, T.W., O'Brien, N., O'Donovan, C., Olde Loohuis, L.M., Oruc, L., Papiol, S., Pardinas, A.F., Perry, A., Pfennig, A., Porichi, E., Potash, J.B., Quested, D., Raj, T., Rapaport, M.H., DePaulo, J.R., Regeer, E.J., Rice, J.P., Rivas, F., Rivera, M., Roth, J., Roussos, P., Ruderfer, D.M., Sanchez-Mora, C., Schulte, E.C., Senner, F., Sharp, S., Shilling, P.D., Sigurdsson, E., Sirignano, L., Slaney, C., Smeland, O.B., Smith, D.J., Sobell, J.L., Soholm Hansen, C., Soler Artigas, M., Spijker, A.T., Stein, D.J., Strauss, J.S., Swiatkowska, B., Terao, C., Thorgeirsson, T.E., Toma, C., Tooney, P., Tsermpini, E.E., Vawter, M.P., Vedder, H., Walters, J.T.R., Witt, S.H., Xi, S., Xu, W., Yang, J.M.K., Young, A.H., Young, H., Zandi, P.P., Zhou, H., Zillich, L., Psychiatry, H.A.-I., Adolfsson, R., Agartz, I., Alda, M., Alfredsson, L., Babadjanova, G., Backlund, L., Baune, B.T., Bellivier, F., Bengesser, S., Berrettini, W.H., Blackwood, D.H.R., Boehnke, M., Borglum, A.D., Breen, G., Carr, V.J., Catts, S., Corvin, A., Craddock, N., Dannlowski, U., Dikeos, D., Esko, T., Etain, B., Ferentinos, P., Frye, M., Fullerton, J.M., Gawlik, M., Gershon, E.S., Goes, F.S., Green, M.J., Grigoroiu-Serbanescu, M., Hauser, J., Henskens, F., Hillert, J., Hong, K.S., Hougaard, D.M., Hultman, C.M., Hveem, K., Iwata, N., Jablensky, A.V., Jones, I., Jones, L.A., Kahn, R.S., Kelsoe, J.R., Kirov, G., Landen, M., Leboyer, M., Lewis, C.M., Li, Q.S., Lissowska, J., Lochner, C., Loughland, C., Martin, N.G., Mathews, C.A., Mayoral, F., McElroy, S.L., McIntosh, A.M., McMahon, F.J., Melle, I., Michie, P., Milani, L., Mitchell, P.B., Morken, G., Mors, O., Mortensen, P.B., Mowry, B., Muller-Myhsok, B., Myers, R.M., Neale, B.M., Nievergelt, C.M., Nordentoft, M., Nothen, M.M., O'Donovan, M.C., Oedegaard, K.J., Olsson, T., Owen, M.J., Paciga, S.A., Pantelis, C., Pato, C., Pato, M.T., Patrinos, G.P., Perlis, R.H., Posthuma, D., Ramos-Quiroga, J.A., Reif, A., Reininghaus, E.Z., Ribases, M., Rietschel, M., Ripke, S., Rouleau, G.A., Saito, T., Schall, U., Schalling, M., Schofield, P.R., Schulze, T.G., Scott, L.J., Scott, R.J., Serretti, A., Shannon Weickert, C., Smoller, J.W., Stefansson, H., Stefansson, K., Stordal, E., Streit, F., Sullivan, P.F., Turecki, G., Vaaler, A.E., Vieta, E., Vincent, J.B., Waldman, I.D., Weickert, T.W., Werge, T., Wray, N.R., Zwart, J.A., Biernacka, J.M., Nurnberger, J.I., Cichon, S., Edenberg, H.J., Stahl, E.A., McQuillin, A., Di Florio, A., Ophoff, R.A., Andreassen, O.A., 2021. Genome-wide association study of more than 40,000 bipolar disorder cases provides new insights into the underlying biology. Nat Genet.

Pardinas, A.F., Holmans, P., Pocklington, A.J., Escott-Price, V., Ripke, S., Carrera, N., Legge, S.E., Bishop, S., Cameron, D., Hamshere, M.L., Han, J., Hubbard, L., Lynham, A., Mantripragada, K., Rees, E., MacCabe, J.H., McCarroll, S.A., Baune, B.T., Breen, G., Byrne, E.M., Dannlowski, U., Eley, T.C., Hayward, C., Martin, N.G., McIntosh, A.M., Plomin, R., Porteous, D.J., Wray, N.R., Caballero, A., Geschwind, D.H., Huckins, L.M., Ruderfer, D.M., Santiago, E., Sklar, P., Stahl, E.A., Won, H., Agerbo, E., Als, T.D., Andreassen, O.A., Baekvad-Hansen, M., Mortensen, P.B., Pedersen, C.B., Borglum, A.D., Bybjerg-Grauholm, J., Djurovic, S., Durmishi, N., Pedersen, M.G., Golimbet, V., Grove, J., Hougaard, D.M., Mattheisen, M., Molden, E., Mors, O., Nordentoft, M., Pejovic-Milovancevic, M., Sigurdsson, E., Silagadze, T., Hansen, C.S., Stefansson, K., Stefansson, H., Steinberg, S., Tosato, S., Werge, T., Consortium, G., Consortium, C., Collier, D.A., Rujescu, D., Kirov, G., Owen, M.J., O'Donovan, M.C., Walters, J.T.R., Consortium, G., Consortium, C., Consortium, G., Consortium, C., 2018. Common schizophrenia alleles are enriched in mutation-intolerant genes and in regions under strong background selection. Nat Genet 50, 381-389.

Pierce, B.L., Ahsan, H., Vanderweele, T.J., 2011. Power and instrument strength requirements for Mendelian randomization studies using multiple genetic variants. Int J Epidemiol 40, 740-752.

Sarwar, N., Butterworth, A.S., Freitag, D.F., Gregson, J., Willeit, P., Gorman, D.N., Gao, P., Saleheen, D., Rendon, A., Nelson, C.P., Braund, P.S., Hall, A.S., Chasman, D.I., Tybjaerg-Hansen, A., Chambers, J.C., Benjamin, E.J., Franks, P.W., Clarke, R., Wilde, A.A., Trip, M.D., Steri, M., Witteman, J.C., Qi, L., van der Schoot, C.E., de Faire, U., Erdmann, J., Stringham, H.M., Koenig, W., Rader, D.J., Melzer, D., Reich, D., Psaty, B.M., Kleber, M.E., Panagiotakos, D.B., Willeit, J., Wennberg, P., Woodward, M., Adamovic, S., Rimm, E.B., Meade, T.W., Gillum, R.F., Shaffer, J.A., Hofman, A., Onat, A., Sundstrom, J., Wassertheil-Smoller, S., Mellstrom, D., Gallacher, J., Cushman, M., Tracy, R.P., Kauhanen, J., Karlsson, M., Salonen, J.T., Wilhelmsen, L., Amouyel, P., Cantin, B., Best, L.G., Ben-Shlomo, Y., Manson, J.E., Davey-Smith, G., de Bakker, P.I., O'Donnell, C.J., Wilson, J.F., Wilson, A.G., Assimes, T.L., Jansson, J.O., Ohlsson, C., Tivesten, A., Ljunggren, O., Reilly, M.P., Hamsten, A., Ingelsson, E., Cambien, F., Hung, J., Thomas, G.N., Boehnke, M., Schunkert, H., Asselbergs, F.W., Kastelein, J.J., Gudnason, V., Salomaa, V., Harris, T.B., Kooner, J.S., Allin, K.H., Nordestgaard, B.G., Hopewell, J.C., Goodall, A.H., Ridker, P.M., Holm, H., Watkins, H., Ouwehand, W.H., Samani, N.J., Kaptoge, S., Di Angelantonio, E., Harari, O., Danesh, J., 2012. Interleukin-6 receptor pathways in coronary heart disease: a collaborative meta-analysis of 82 studies. Lancet 379, 1205-1213.

Schizophrenia Working Group of the Psychiatric Genomics, C., 2014. Biological insights from 108 schizophrenia-associated genetic loci. Nature 511, 421-427.

Swerdlow, D.I., Holmes, M.V., Kuchenbaecker, K.B., Engmann, J.E., Shah, T., Sofat, R., Guo, Y., Chung, C., Peasey, A., Pfister, R., Mooijaart, S.P., Ireland, H.A., Leusink, M., Langenberg, C., Li, K.W., Palmen, J., Howard, P., Cooper, J.A., Drenos, F., Hardy, J., Nalls, M.A., Li, Y.R., Lowe, G., Stewart, M., Bielinski, S.J., Peto, J., Timpson, N.J., Gallacher, J., Dunlop, M., Houlston, R., Tomlinson, I., Tzoulaki, I., Luan, J., Boer, J.M., Forouhi, N.G., Onland-Moret, N.C., van der Schouw, Y.T., Schnabel, R.B., Hubacek, J.A., Kubinova, R., Baceviciene, M., Tamosiunas, A., Pajak, A., Topor-Madry, R., Malyutina, S., Baldassarre, D., Sennblad, B., Tremoli, E., de Faire, U., Ferrucci, L., Bandenelli, S., Tanaka, T., Meschia, J.F., Singleton, A., Navis, G., Mateo Leach, I., Bakker, S.J., Gansevoort, R.T., Ford, I., Epstein, S.E., Burnett, M.S., Devaney, J.M., Jukema, J.W., Westendorp, R.G., Jan de Borst, G., van der Graaf, Y., de Jong, P.A., Mailand-van der Zee, A.H., Klungel, O.H., de Boer, A., Doevendans, P.A., Stephens, J.W., Eaton, C.B., Robinson, J.G., Manson, J.E., Fowkes, F.G., Frayling, T.M., Price, J.F., Whincup, P.H., Morris, R.W., Lawlor, D.A., Smith, G.D., Ben-Shlomo, Y., Redline, S., Lange, L.A., Kumari, M., Wareham, N.J., Verschuren, W.M., Benjamin, E.J., Whittaker, J.C., Hamsten, A., Dudbridge, F., Delaney, J.A., Wong, A., Kuh, D., Hardy, R., Castillo, B.A., Connolly, J.J., van der Harst, P., Brunner, E.J., Marmot, M.G., Wassel, C.L., Humphries, S.E., Talmud, P.J., Kivimaki, M., Asselbergs, F.W., Voevoda, M., Bobak, M., Pikhart, H., Wilson, J.G., Hakonarson, H., Reiner, A.P., Keating, B.J., Sattar, N., Hingorani, A.D., Casas, J.P., Consortium, I.-R.M.R.A.I.R.M., 2012. The interleukin-6 receptor as a target for prevention of coronary heart disease: a mendelian randomisation analysis. Lancet 379, 1214-1224.

Verbanck, M., Chen, C.Y., Neale, B., Do, R., 2018. Detection of widespread horizontal pleiotropy in causal relationships inferred from Mendelian randomization between complex traits and diseases. Nat Genet 50, 693-698.

Wray, N.R., Ripke, S., Mattheisen, M., Trzaskowski, M., Byrne, E.M., Abdellaoui, A., Adams, M.J., Agerbo, E., Air, T.M., Andlauer, T.M.F., Bacanu, S.A., Bækvad-Hansen, M., Beekman, A.F.T., Bigdeli, T.B., Binder, E.B., Blackwood, D.R.H., Bryois, J., Buttenschøn, H.N., Bybjerg-Grauholm, J., Cai, N., Castelao, E., Christensen, J.H., Clarke, T.K., Coleman, J.I.R., Colodro-Conde, L., Couvy-Duchesne, B., Craddock, N., Crawford, G.E., Crowley, C.A., Dashti, H.S., Davies, G., Deary, I.J., Degenhardt, F., Derks, E.M., Direk, N., Dolan, C.V., Dunn, E.C., Eley, T.C., Eriksson, N., Escott-Price, V., Kiadeh, F.H.F., Finucane, H.K., Forstner, A.J., Frank, J., Gaspar, H.A., Gill, M., Giusti-Rodríguez, P., Goes, F.S., Gordon, S.D., Grove, J., Hall, L.S., Hannon, E., Hansen, C.S., Hansen, T.F., Herms, S., Hickie, I.B., Hoffmann, P., Homuth, G., Horn, C., Hottenga, J.J., Hougaard, D.M., Hu, M., Hyde, C.L., Ising, M., Jansen, R., Jin, F., Jorgenson, E., Knowles, J.A., Kohane, I.S., Kraft, J., Kretzschmar, W.W., Krogh, J., Kutalik, Z., Lane, J.M., Li, Y., Lind, P.A., Liu, X., Lu, L., MacIntyre, D.J., MacKinnon, D.F., Maier, R.M., Maier, W., Marchini, J., Mbarek, H., McGrath, P., McGuffin, P., Medland, S.E., Mehta, D., Middeldorp, C.M., Mihailov, E., Milaneschi, Y., Milani, L., Mill, J., Mondimore, F.M., Montgomery, G.W., Mostafavi, S., Mullins, N., Nauck, M., Ng, B., Nivard, M.G., Nyholt, D.R., O'Reilly, P.F., Oskarsson, H., Owen, M.J., Painter, J.N., Pedersen, C.B., Pedersen, M.G., Peterson, R.E., Pettersson, E., Peyrot, W.J., Pistis, G., Posthuma, D., Purcell, S.M., Quiroz, J.A., Qvist, P., Rice, J.P., Riley, B.P., Rivera, M., Saeed Mirza, S., Saxena, R., Schoevers, R., Schulte, E.C., Shen, L., Shi, J., Shyn, S.I., Sigurdsson, E., Sinnamon, G.B.C., Smit, J.H., Smith, D.J., Stefansson, H., Steinberg, S., Stockmeier, C.A., Streit, F., Strohmaier, J., Tansey, K.E., Teismann, H., Teumer, A., Thompson, W., Thomson, P.A., Thorgeirsson, T.E., Tian, C., Traylor, M., Treutlein, J., Trubetskoy, V., Uitterlinden, A.G., Umbricht, D., Van der Auwera, S., van Hemert, A.M., Viktorin, A., Visscher, P.M., Wang, Y., Webb, B.T., Weinsheimer, S.M., Wellmann, J., Willemsen, G., Witt, S.H., Wu, Y., Xi, H.S., Yang, J., Zhang, F., Arolt, V., Baune, B.T., Berger, K., Boomsma, D.I., Cichon, S., Dannlowski, U., de Geus, E.C.J., DePaulo, J.R., Domenici, E., Domschke, K., Esko, T., Grabe, H.J., Hamilton, S.P., Hayward, C., Heath, A.C., Hinds, D.A., Kendler, K.S., Kloiber, S., Lewis, G., Li, Q.S., Lucae, S., Madden, P.F.A., Magnusson, P.K., Martin, N.G., McIntosh, A.M., Metspalu, A., Mors, O., Mortensen, P.B., Müller-Myhsok, B., Nordentoft, M., Nöthen, M.M., O'Donovan, M.C., Paciga, S.A., Pedersen, N.L., Penninx, B.W.J.H., Perlis, R.H., Porteous, D.J., Potash, J.B., Preisig, M., Rietschel, M., Schaefer, C., Schulze, T.G., Smoller, J.W., Stefansson, K., Tiemeier, H., Uher, R., Völzke, H., Weissman, M.M., Werge, T., Winslow, A.R., Lewis, C.M., Levinson, D.F., Breen, G., Børglum, A.D., Sullivan, P.F., eQTLGen, 23andMe, Consortium, M.D.D.W.G.o.t.P.G., 2018. Genome-wide association analyses identify 44 risk variants and refine the genetic architecture of major depression. Nat Genet 50, 668-681.
